# Gut microbiota modulate neurobehavior through changes in brain insulin sensitivity and metabolism

**DOI:** 10.1038/s41380-018-0086-5

**Published:** 2018-06-18

**Authors:** Marion Soto, Clémence Herzog, Julian A. Pacheco, Shiho Fujisaka, Kevin Bullock, Clary B. Clish, C. Ronald Kahn

**Affiliations:** 1 Section of Integrative Physiology and Metabolism, Joslin Diabetes Center and Department of Medicine, Harvard Medical School, Boston, MA 02215 USA; 2 Broad Institute of MIT and Harvard, Cambridge, MA 02142 USA; 3 First Department of Internal Medicine, University of Toyama, Toyama, 930-0194 Japan

## Abstract

Obesity and diabetes in humans are associated with increased rates of anxiety and depression. To understand the role of the gut microbiome and brain insulin resistance in these disorders, we evaluated behaviors and insulin action in brain of mice with diet-induced obesity (DIO) with and without antibiotic treatment. We find that DIO mice have behaviors reflective of increased anxiety and depression. This is associated with decreased insulin signaling and increased inflammation in in the nucleus accumbens and amygdala. Treatment with oral metronidazole or vancomycin decreases inflammation, improves insulin signaling in the brain and reduces signs of anxiety and depression. These effects are associated with changes in the levels of tryptophan, GABA, BDNF, amino acids, and multiple acylcarnitines, and are transferable to germ-free mice by fecal transplant. Thus, changes in gut microbiota can control brain insulin signaling and metabolite levels, and this leads to altered neurobehaviors.

## Introduction

Over the past decade there has been a mounting body of evidence that gut microbiota can modulate host physiology in mice, humans and other species [1–6]. Many factors can affect the composition of the gut microbiome, including colonization at time of birth, changes in the diet, and exposure to antibiotics [7–10]. In both humans and mice, feeding a high fat diet (HFD) or over-eating due to a genetic mutation in the leptin axis induces robust alterations in gut microbial flora, reducing bacterial diversity and altering the overall bacterial composition [11, 12]. These shifts in microbial composition can affect the host’s physiology, inducing systemic insulin resistance and modifying glucose homeostasis and the immune response [1, 8]. The modifications in gut microbiota composition contribute to these metabolic changes as demonstrated by the fact that some of the metabolic abnormalities associated with obesity, including over-eating and weight gain, can be reproduced in germ-free (GF) mice by colonization with gut microbiota from obese mice or obese humans [4, 5]. In addition, we [8] and others [13–15] have shown that treatment with prebiotics, probiotics or antibiotics, which modulate the gut microbiome, can reduce insulin resistance and inflammation in peripheral organs, such as liver, fat and muscle, in mice models.

In addition to metabolic abnormalities, both obesity and diabetes are associated with increased risk of neuropsychiatric and mood disorders, including poorer cognitive performance, and increased rates of depression, anxiety and dementia [16–18]. These can be reproduced in mouse models by induction of diet-induced obesity (DIO) [19–21]. One potential contributor to these neurobehavioral abnormalities is the gut microbiome [22]. Other studies have shown that modifying the gut microbiota of conventional mice by the use of prebiotics [23] and probiotics [24] can improve neurobehavior and that in addition to the abnormalities in gut development and metabolism which can be reversed by microbial re-colonization, GF-mice have decreased anxiety-like behavior that are also reversed by microbiome colonization [25, 26].

Here, we investigate the link between gut microbiota and brain function in a context of obesity and metabolic syndrome using mice challenged with a HFD, without or with treatment by vancomycin or metronidazole, to further modify the microbiome. We find that DIO mice exhibit insulin resistance in brain and multiple depressive-like and anxiety-like behaviors, and these are improved by antibiotic treatment. These changes are transferrable to germ-free mice, and are associated with modifications in the levels of neurotransmitters and other metabolites, which can affect brain function.

## Methods

### Animals

Six-week-old male *C57BL/6J* mice were purchased from Jackson Laboratory and maintained on either a normal chow containing 22% of calories from fat, 23% from protein, and 55% from carbohydrates (Mouse diet 9F 5020; PharmaServ) or a high-fat diet (Open Source Diet, D12492; Research Diets) containing 60% of calories from fat, 20% from protein, and 20% from carbohydrates for 6 weeks. During the last 2 weeks, some of the HFD mice were treated with vancomycin or metronidazole (1 g/L in the drinking water).

For germ-free (GF) experiments, cecal contents from mice that received the treatment described above were collected immediately after euthanasia, suspended in PBS, and filtered through a 40 μm cell strainer. Bacterial transfer was performed by gastric gavage with 200 μl of diluted cecal contents. Six-week-old male GF-*C57BL/6J* mice (obtained from the gnotobiotic core facility of Brigham and Women’s Hospital) were given chow or HFD (double irradiated D1249ii, Research Diets) for 4 weeks, and then gavaged with cecum contents from donor mice. The colonized mice were continuously fed chow or HFD for 2 more weeks.

To determine the effect of removing the antibiotic treatment, mice were fed chow or HFD for 11 weeks without or with antibiotics (as described above) from week 5 to week 7, and then switched to normal water. All mice were housed at 22 °C on a 12 h light/dark cycle. All animal studies were approved by the IACUC of Joslin Diabetes Center (# 97-05) and Harvard Medical School (# 05131) and were in accordance with NIH guidelines.

### 16S rRNA sequence analysis

Six-week-old male C57BL/6 J mice were treated with either placebo, vancomycin (1 g/L) or metronidazole (1 g/L) in drinking water then started on HFD (or continued on chow) from age 7 to 11 weeks. The mice were fasted for 2 h, anesthetized with isoflurane and cecum was collected. DNA was extracted from cecum samples using a MoBio Fecal DNA extraction kit (MoBio Laboratories Inc., Carlsbad, CA). A multiplexed amplicon library covering the 16 S rDNA gene V4 region was generated from DNA extracted samples, and reads were generated from the amplicon library and clustered into Operational Taxonomic Units (OTUs) (MiSeq instrument–average of 27,331.5 reads per sample. Differences in microbial community structure were visualized using phylogenetic methods. The number of OTUs per sample were then scaled so each sample had the same mean, filtered to only include OTUs that were present at 0.1% of the total counts in at least 3 samples, log-transformed, and plotted in PCA space using the R software. PCA plots, as well as representation of bacterial communities at the phylum levels are described in another paper [27]. 16S rRNA datasets have been deposited in Sequence Read Archive (SRA) database (accession number: SRP132006).

### Metabolic studies

Food intake was estimated by weekly measures of weight differential of cups containing the diet. Body weight was measured every week. Locomotor activity was measured at ambient temperature using a Comprehensive Lab Animal Monitoring System (Columbus Instruments). Oral glucose tolerance tests (2 g dextrose/kg body weight) were performed by gavage in mice fasted for 4 h. Tail vein blood glucose levels were measured using Infinity glucose monitors (US Diagnostics). To assess insulin signaling in vivo, 5 U insulin (Humulin R, Eli Lilly) was injected via the inferior vena cava. Samples were collected 10 min later and immediately frozen in liquid nitrogen. Insulin and leptin were measured by ELISA (Crystal Chem and Invitrogen).

### Open field exploration

The open field test is widely used to measure anxiety-like in rodents [20, 28]. The activity of the mouse was recorded by an overhead video camera placed on top of an open field box (57 × 37 × 31 cm) for 5 min. ANY Maze video tracking software (Stoelting) was used to analyze the number of entries, latency to enter and duration of time spent in the center arena, as described before [28]. For all behavioral tests, data recording and potential subsequent analysis were blinded.

### Novelty-suppressed feeding

NSF measures a rodent’s aversion to eating in a novel environment. This test assesses stress-induced anxiety by measuring the latency of an animal to approach and eat a familiar food in an aversive environment. Mice were fasted overnight (~15 h) and placed in the open field box with a white disc (15 cm diameter) in the center in which rested a food pellet of their usual diet (either chow or HFD according to the groups) for 10 min. The latency to start feeding was measured to assess the anxiety-related behavior.

### Dark-light test

This test is a measure of anxiety-related behavior, based on the innate aversion of rodents to brightly illuminated areas. The apparatus (44 × 21 × 21 cm) contains two chambers of equal size, one bright and the other dark. The animals were initially placed in the light side. Transitions between sides and the time spent in each compartment were recorded for 5 min by a camera and analyzed with AnyMaze software.

### Marble burying

Marble burying is an elicited repetitive behavior in rodents notably observed in models of autism [29]. Mice were transferred to a new housing cage with 5 cm wood chip bedding. 15 glass marbles (16 mm diameter) were aligned equidistantly in a 5 × 3 design in the testing cage. The number of marbles buried (min. 1:2 their depth) in 30 min was recorded.

### Cytokine ELISAs

Frozen brain tissue were homogenized in RIPA buffer containing protease inhibitor cocktail (ThermoFisher, Pittsburgh, PA), centrifuged for 15 min at 13000 r.p.m. and diluted into PBS (1:2). TNFα and IL-1β ELISAs (eBioscience) were performed according to manufacturer’s instructions. The amount of protein in each well was quantified using protein microassay (Bio-Rad).

### Protein analysis

Frozen tissues were lysed in ice-cold buffer (RIPA buffer complemented with 0.1% SDS and 1× protease and phosphatase inhibitor cocktail (Biotool)). Protein concentrations were determined using the Pierce BCA protein assay reagent (Thermo Scientific). Lysates (15-20 μg) were separated on NuPage 4–12% polyacrylamide gels (ThermoFisher) and transferred to nitrocellulose membranes (ThermoFisher). Antibodies against phospho-Akt (S473) (#4060, 1:1000), Akt (#4685, 1:1000), phospho-Creb (S133) (#9198, 1:600), Creb (#9197, 1:1000), phopsho-DARP32 (T75) (#2301, 1:1000), DARP32 (#2306, 1:1000), ΔFosB (#9890, 1:1000), phospho-ERK1/2 (T202-Y204) (#4370, 1:1000), ERK1/2 (#9102, 1:1000), GAPDH (#5174, 1:2000), phospho-IR/IGF1R (#3024, 1:500), IRβ (#3020, 1:1000) and TH (#2792, 1:1000) were purchased from Cell Signaling Technologies. Anti-β-actin (#1616-HRP, 1:10 000) and BDNF (#546, 1:500) antibodies were from Santa-Cruz. Cd11b (MCA711G, 1:1000) antibody was from Bio-Rad. Anti-DAT (MAB369, 1:5000), phospho-IRS-1 (Y608) (09432, 1:1000) and IRS-1 (06248, Millipore, 1:1000) were from Millipore. Anti-GFAP (#7260, 1:1000) was from Abcam and anti-Iba1 (01620001, 1:1000) was purchased from Wako. The appropriate secondary antibodies conjugated to horseradish peroxidase (HRP) were from Santa Cruz Biotechnology (1:7000). Membranes were visualized with Immobilon Western HRP Substrate (Millipore, Billerica, MA). Quantification was performed using ImageJ software.

### Immunostaining

Mice were anesthetized with an intraperitoneal (i.p.) injection of Avertin (300 mg/kg) and transcardially perfused with PBS followed by 4 % paraformaldehyde. Brains were dissected and fixed in 4% paraformaldehyde overnight, cryoprotected in 15% (w/v) then 30% sucrose, and frozen in OCT compound (Tissue-Tek). Serial 30 μm coronal sections were generated using a cryostat. Free-floating sections were washed, blocked (0.2% Triton-X-100 + 5% normal goat serum (NGS) in PBS) and stained with anti-Cd11b (1:50, rat, MCA711G, Bio-Rad) and anti-rabbit GFAP (1:500; rabbit, #7260, Abcam) antibodies diluted in PBS containing 0.1 Triton X-100 + 1% NGS overnight at 4 °C. Sections were then washed and subsequently stained with secondary antibodies (goat anti-rabbit Alexa Fluor 488 and goat anti-rat Alexa Fluor 546, 1:500, ThermoFisher Scientific). Sections were mounted with SlowFade Diamond mountant containing DAPI (Life Technologies) and imaged with a 10× objective on a Zeiss LSM710-Duo confocal microscope.

### qRT-PCR

RNA was extracted from tissues with an RNeasy minikit (Qiagen), and complementary DNA (cDNA) was synthesized with a high-capacity cDNA reverse-transcription kit (Applied Biosystems). Real-time qPCR was performed using SYBR Green (Bio-Rad) with specific primers (Supplementary Table 1) on an ABI Prism 7900HT sequence detection system (Applied Biosystems). Amplification of specific transcripts was confirmed by analyzing melting curve profiles at the end of each PCR. All expression data were normalized to TATA-binding protein (TBP) expression using the relative standard curve method.

### Metabolomic and bioinformatic analysis

Frozen brain tissue samples were homogenized in 4 volumes of HPLC water using a TissueLyser II (Qiagen). Polar metabolites were profiled using liquid chromatography tandem mass spectrometry (LC-MS). Positive ionization mode data were acquired using multiple reaction monitoring and retention times, mass transitions, and collision energies, determined with authentic reference standards (Supplementary Table 2). Additional information is described in Supplemental Experimental Procedures. Analysis was done in R/Bioconductor. To preprocess the data, metabolites that had missing values in more than 80% of the samples were filtered out, missing values were imputed with half of the minimums of the metabolite’s intensity, and all metabolite intensities were log_2_ transformed. Between group comparisons per metabolite were analyzed accounting for the mean-variance trend and sample-specific weights with the limma package [30]. PCA plots were calculated with the prcomp function.

### Statistical analyzes

All data are presented as mean ± SEM. Data were analyzed by one-way ANOVA (two-way ANOVA was not feasible due to unbalanced design) to look for an effect of the diet (Chow vs. HFD groups) or antibiotic treatment (HFD vs. HFD + Antibiotics) with repeated measures when necessary. This was followed by t-tests to examine pre-planned comparisons of interest. N indicates the number of animals per group. A *p*-value lower than 0.05 was considered statistically significant.

### Data availability

All data and original codes used in this study are available from the corresponding author on reasonable request. The complete data set of metabolites in brain and plasma and their response to diet and antibiotics is available at Metabolomics Workbench (http://www.metabolomicsworkbench.org).

## Results

### Effect of HFD and antibiotic treatment on gut microbial communities

To assess the effects of diet and antibiotics on the gut microbiome composition, 6-week-old mice were divided into four groups. Two were given regular drinking water (placebo), one drinking water containing vancomycin (1 g/L), which kills gram positive organisms, and the fourth drinking water containing metronidazole (1 g/L), which kills anaerobes. One week later both antibiotic-treated groups and one of the two control groups were challenged with a high fat diet (60% fat by calories) for 4 weeks, while the second control group remained on normal chow (22% fat by calories). 16S rRNA sequence analysis of cecal contents showed large differences of the composition of bacterial communities between the different groups [27] as represented in a principal component analysis, which distinctly separated in all four groups (Supplementary Fig. 1a). While the Shannon Index was not different between chow-fed and HFD-fed mice, entropy was significantly decreased by vancomycin and tended to be decreased by metronidazole (Supplementary Fig. 1b), consistent with a decrease in diversity of microbial species in the antibiotic-treated groups. Analysis of the microbiota at the class level demonstrated the expected reduction in Bacteriodetes Bacteroidia and increase in Firmicutes (Clostridia) and Verrucomicrobiae in response to HFD. Treatment with either antibiotic resulted in elimination of many classes of bacteria, with the remaining bacteria being primarily Firmicutes of the Bacillus class, i.e., Bacillales and Lactobacillales (Supplementary Fig. 1c). In a more detailed analysis of these data, we show that these changes in gut microbial composition are associated with different predicted functions of the microbiome and affect levels of a large number of metabolites in both cecum and blood of host, with some correlating with insulin resistance [27].

One read-out of changing gut microbiota composition is cecum size, which has been shown to be increased in GF-mice and decreased in these mice after microbiota transfer [31]. Interestingly, in HFD-fed DIO mice, cecum weight was reduced by ~ 50%, and this was restored, or even increased above control levels, by antibiotic treatment (Supplementary Fig. 2a). These changes were reversible and were not observed 4 weeks after discontinuation of antibiotics (Supplementary Fig. 2b). The changes in cecum size were a measure of the changing microbiome and could be reproduced, and even magnified, in GF-mice receiving gut microbiota transfer from HFD and HFD-antibiotic-treated mice (Supplementary Fig. 2c).

### Effect of antibiotic treatment on metabolism in HFD-fed mice

**Fig. 1:**
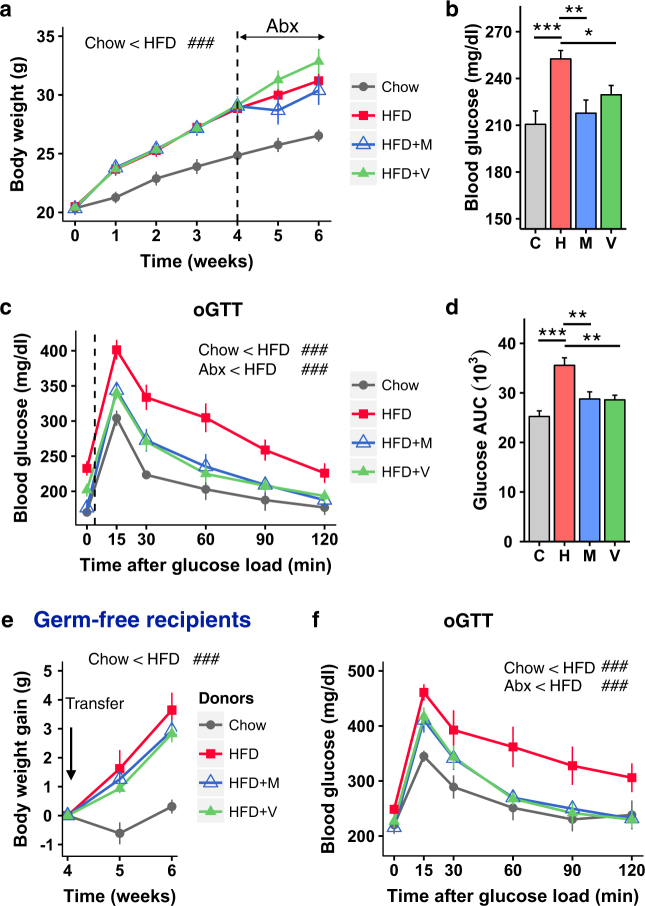
Effect of antibiotic treatment on metabolism in HFD-fed mice. **a** Body weight gain of mice on Chow or HFD with or without Abx treatment (metronidazole or vancomycin (*n* = 10/group). Time 0 is the beginning of the HFD and the dashed line represents the beginning of Abx treatment. **b** Blood glucose levels in the random fed state of mice on a Chow or HFD with or without Abx treatment (*n* = 10/group). **c** OGTT performed in mice on a Chow or HFD with or without Abx treatment (*n* = 9/group). **d** AUC of blood glucose levels during an OGTT (*n* = 9/group). **e** Weight gain of chow or HFD recipient GF-mice after bacterial transfer from mice treated with chow/HFD with or without Abx (*n* = 8-9/group). **f** OGTT of chow or HFD recipient GF-mice 2 weeks after bacterial transfer from mice treated with chow/HFD with or without Abx (*n* = 8/group). C chow, H HFD, M HFD + metronidazole, V HFD + vancomycin. Data are shown as mean ± SEM. ^##^*P* < 0.01, and ^###^*P* < 0.001 by repeated measure ANOVA. *P < 0.05, **P < 0.01, and ***P < 0.001 by ANOVA, followed by pre-planned *t*-tests

It is known that even shorter-term changes in diet can induce changes in the microbiome and metabolism. Thus, for the metabolic and behavioral studies, a second protocol was developed in which 6-week-old male *C57BL/6J* mice were fed either normal chow or a HFD for six weeks, and two subgroups were given drinking water with either vancomycin or metronidazole during the last two weeks of the HFD challenge. As with the longer-term diet study [27], HFD-fed mice had higher caloric intake, showed greater body weight gain and higher blood glucose levels than chow-fed mice (Fig. 1a,b and Supplementary Fig. 2d). Treatment of the HFD-fed mice with vancomycin or metronidazole during the last two weeks of the HFD challenge reduced the level of hyperglycemia to that of chow-fed controls without impacting body weight gain (Fig. 1a,b). Oral glucose tolerance testing (oGTT) of HFD-fed mice revealed marked glucose intolerance, which was significantly improved in the groups that received either antibiotic compared to those on HFD alone (Fig. 1c,d). As anticipated, HFD-feeding increased circulating leptin and insulin levels (Supplementary Fig. 2e,f). While antibiotic treatment had no significant effect on leptin levels, insulin levels tended to be lower in HFD-antibiotic-treated mice, indicating reduced insulin resistance (Supplementary Fig. 2f). When antibiotics were discontinued and the mice followed for an additional 4 weeks on HFD, blood glucose levels during an oral GTT in mice that had received antibiotics reversed and rebounded to even higher levels than those of HFD-fed mice that had never been treated with antibiotics (Supplementary Fig. 2g).

To determine to what extent the improved metabolic phenotype observed in antibiotic-treated HFD-fed mice was secondary to changes in gut microbiota, we performed microbiota transfer from the cecum of chow- and HFD-fed donor mice (including those with or without vancomycin- or metronidazole-treatment) into germ-free (GF) C57BL/6 J recipient mice that had been previously started on either irradiated chow or HFD and then continued on the same diets for two additional weeks after transplant, but with no antibiotic treatment. HFD-fed GF-mice that received bacteria from HFD-fed donor mice gained more weight than chow-fed GF-mice that received bacteria from chow-fed donor mice (Fig. 1e) and displayed higher fasting blood glucose levels and markedly impaired glucose intolerance during an oGTT (Fig. 1f). These mice also had a 2.5-fold increase in fasting insulin levels indicative of insulin resistance, although this did not quite reach statistical significance due to inter-animal variability (Supplementary Fig. 2h). HFD-fed GF-mice receiving microbiota from the HFD-fed antibiotic-treated donor mice showed weight gain similar to that of mice receiving microbiota from HFD-fed, non-antibiotic-treated donors, but has significantly improved fasting insulin levels, blood glucose levels and significantly improved glucose tolerance compared with mice colonized with HFD-only donors (Fig. 1e,f and Supplementary Fig. 2h). Thus, the improvements in the metabolic status by antibiotics were, in large part, transferable via transplant of gut microbiota, despite the fact that recipient mice never received antibiotics. Together, these data demonstrate that antibiotics ameliorate the HFD-associated alterations in the gut and in metabolism, and that these changes are transferrable to germ-free mice and do not persist after antibiotic removal, consistent with a role of modifications in gut microbiota composition in determining these effects.

### Antibiotic treatment reverses HFD-induced depressive and anxiety-like behaviors

**Fig. 2:**
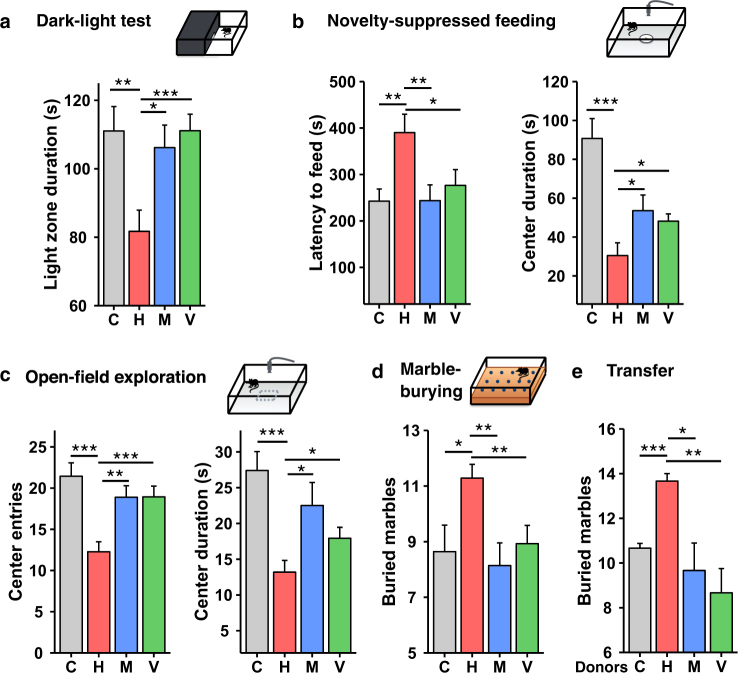
Antibiotic treatment reverses HFD-induced depressive and anxiety-like behaviors. **a** Time spent in light compartment during light/dark box test of mice on a Chow or HFD with or without Abx treatment (*n* = 18/group). **b** Assessment of conflict-based anxiety as latency to feed and time spent in the center zone during novelty suppressed feeding test (*n* = 18/group). **c** Number of entries and time spent in the center during open-field exploration (*n* = 18/group). **d** Assessment of anxiety as number of buried marbles during a marble-burying task (*n* = 14/group). **e** Assessment of anxiety as number of buried marbles during a marble-burying task of chow or HFD recipient GF-mice 2 weeks after bacterial transfer from mice treated with chow/HFD with or without Abx (*n* = 6/group). C chow, H HFD, M HFD + metronidazole, V HFD + vancomycin. Data are shown as mean ± SEM. *P < 0.05, **P < 0.01, and ***P < 0.001 by ANOVA, followed by pre-planned *t*-tests

Obesity in humans is associated with an increased risk of anxiety and depression [18, 32], and DIO mice have also been shown to have altered behaviors reflective of increased depression and anxiety [20, 21]. To determine the role of the gut microbiome on these behaviors, mice on chow or HFD for six weeks without or with antibiotics for the final two weeks were subjected to a panel of behavioral tests at the end of the study period. Using a dark/light box, we found that HFD-fed mice ventured into the light compartment 26% less of the time than control mice (Fig. 2a), consistent with increased anxiety, and this was reversed by treatment of HFD-fed mice with either vancomycin or metronidazole. To further assess anxiety, mice were subjected to a novelty-suppressed feeding test, in which fasted mice are placed in an open-field box containing food pellets in its center, but on a white paper disc, which creates anxiety to enter the center and feed. In HFD-fed mice, the latency to feed was increased by 1.6-fold (Fig. 2b). As a result, there was a significant decrease in the time spent in the center of the box (Fig. 2b). Antibiotic treatment significantly reversed these behavioral changes to the level of chow-fed controls (Fig. 2b). In an open field exploration test, HFD-fed mice also had signs of increased anxiety with a decreased number of entries and a decreased time spent in the center of the arena (Fig. 2c). Treatment of HFD-fed mice with either antibiotic also reversed these changes, with an increased number of entries and time spent in the center of the box (Fig. 2c). In this test, HFD-fed mice also traveled 23% less distance than chow-fed mice (Supplementary Fig. 3a), and this decrease was reversed by antibiotic treatment, despite no effect on body weight and no change in overall spontaneous locomotor activity levels between HFD and HFD plus antibiotics treated mice as measured in metabolic cages (Supplementary Fig. 3b). Thus, the difference in behavior between HFD and chow-fed controls was not simply due to higher body mass. Like the metabolic changes, the improved behavioral phenotypes observed in the open field test in HFD-fed mice that had been treated with antibiotics returned to DIO levels within four weeks after discontinuation of the antibiotics (Supplementary Fig. 3c).

Finally, we performed a marble burying test which measures the propensity of mice to engage in a digging behavior and is increased in models of anxiety [33]. HFD-fed mice displayed a 1.3-fold increase in marble burying compared to controls, and this was reversed toward chow-fed levels in the antibiotic-treated HFD mice (Fig. 2d). To confirm that these changes in neurobehavior were in fact due to changes in gut microbiota, we assessed marble burying activity in GF-mice, fed chow or HFD, that had been colonized with cecal microbiota from chow, HFD and HFD plus antibiotics donors. Like the donor mice, HFD-fed GF-mice colonized with microbiota from HFD-fed mice showed an increase in marble burying activity compared to chow-fed controls, and this was not observed in recipient mice receiving microbiota from antibiotic-treated HFD-fed donors (Fig. 2e). Thus, HFD-fed mice demonstrate multiple depressive- and anxiety-like behaviors that are ameliorated by either vancomycin or metronidazole treatment and return when the antibiotic treatment is discontinued. They are also transmitted to GF recipient mice by cecal microbiota transfer, indicating a role of the gut microbiome in these behavioral changes.

### Antibiotic treatment ameliorates HFD-induced insulin resistance in the brain

**Fig. 3:**
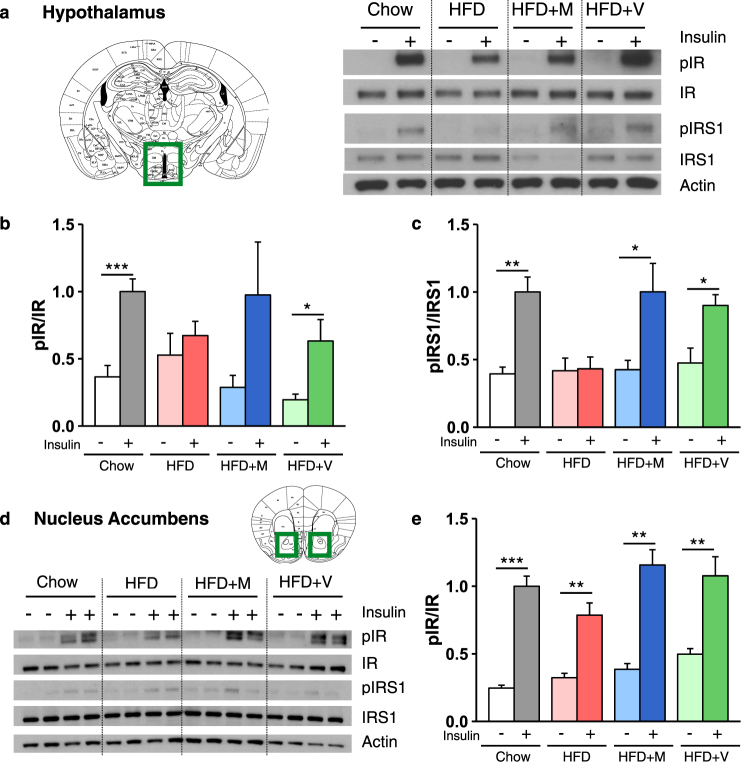
Antibiotic treatment ameliorates HFD-induced insulin resistance in the brain. **a**, **d** Representative western blots of insulin signaling in hypothalamic (**a**) and nucleus accumbens (Nacc) (**d**) extracts from chow, HFD-fed mice and HFD-fed mice treated with metronidazole or vancomycin, after injection of saline or insulin. Actin serves as a loading control. **b**, **c**, **e** Quantification of IR and IRS-1 phosphorylation (pIR and pIRS-1) normalized by total protein levels in the hypothalamus (**b**, **c**, *n* = 5-8/group) and Nacc (**e**, *n* = 5-8/group). M metronidazole, V vancomycin. Data are shown as mean ± SEM, normalized to chow-fed mice stimulated with insulin. *P < 0.05, **P < 0.01, and ***P < 0.001 by pre-planned t-tests

We have previously shown that loss of insulin signaling in brain of mice by tissue specific knockout of the insulin receptor is associated with neurobehavior abnormalities, especially as mice age [28]. We [10] and others [34] have also demonstrated that HFD feeding can induce both systemic and brain insulin resistance in mice. To understand the role of altered insulin signaling in the context of HFD and antibiotic treatment, we assessed insulin receptor (IR) and insulin receptor substrate 1 (IRS-1) phosphorylation in brain regions in vivo, following injection of insulin into the vena cava. We focused on the hypothalamus, a major center in control of peripheral metabolism, and the nucleus accumbens (Nacc), which plays a central role in reward behavior. Following insulin injection, phosphorylation of IR and IRS-1, expressed as the ratio to IR and IRS-1 proteins, was increased by ~2.5-fold in the hypothalamus of chow-fed mice within 10 min (Fig. 3a-c). By contrast, there was little or no increase in the ratio of pIR/IR or pIRS-1/IRS-1 in HFD-fed mice following insulin injection, indicating central insulin resistance. In HFD-fed mice treated with either metronidazole or vancomycin, the ability of insulin to stimulate IR and IRS-1 phosphorylation was returned to near normal (Fig. 3a-c). A similar decrease and rescue of IR and IRS-1 phosphorylation was observed in the Nacc (Fig. 3d,e).

**Fig. 4:**
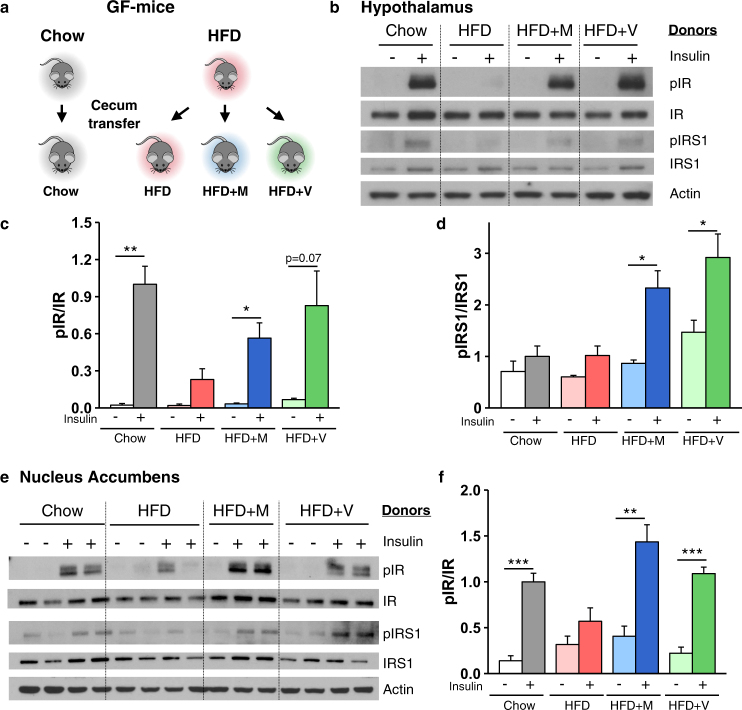
Gut microbiota directly modulates brain insulin signaling. **a** Schematic of the experimental design. Chow or HFD recipient GF-mice were colonized with caecum content of mice treated with chow/HFD with or without Abx (metronidazole (HFD + M) or vancomycin (HFD + V)). **b**, **e** Representative western blots of insulin signaling in hypothalamus (**b**) and Nacc (**e**) from GF-mice colonized with gut microbiota from chow, HFD-fed mice and HFD-fed mice treated with antibiotics, 10 min after vena cava injection of saline or 5 U insulin. Actin served as a loading control. **c**, **d**, **f** Quantification of IR and IRS-1 phosphorylation (pIR and pIRS-1) normalized by total protein levels in the hypothalamus (**c**, **d**, *n* = 4/group) and Nacc (**f**, *n* = 4/group). M metronidazole, V vancomycin. Data are shown as mean ± SEM, normalized to GF-mice colonized with gut microbiota from chow-fed mice, stimulated with insulin. *P < 0.05, **P < 0.01, and ***P < 0.001 by pre-planned *t*-tests

To determine if these changes in brain insulin action were related to changes in the gut microbiome, we performed insulin signaling in GF-mice receiving either chow or HFD and colonized with cecal contents from donor mice treated with chow or HFD, with or without antibiotics (Fig. 4a). As in the donor mice, insulin receptor phosphorylation in the hypothalamus was stimulated 44-fold by peripheral insulin injection in mice colonized with microbiota from chow-fed animals, and this was almost completely lost in HFD-fed GF-mice receiving microbiota from HFD donors (Fig. 4b,c). As in the donor mice, HFD-fed GF-mice colonized with cecal contents from either metronidazole or vancomycin treated HFD mice showed robust insulin stimulation (Fig. 4b,c). IRS-1 phosphorylation showed parallel changes (Fig. 4b,d). Similar changes in IR and IRS-1 phosphorylation were observed in the Nacc (Fig. 4e,f) and in the amygdala, which controls emotional reactions and cognition (Supplementary Fig. 4a-c). Together, these data show that HFD-fed mice display insulin resistance in the brain, which is improved by antibiotic-induced changes in gut microbiota, and these responses are transferred to GF-mice by fecal transplant.

### Antibiotic treatment ameliorates HFD-induced inflammation and BDNF signaling in the nucleus accumbens

**Fig. 5:**
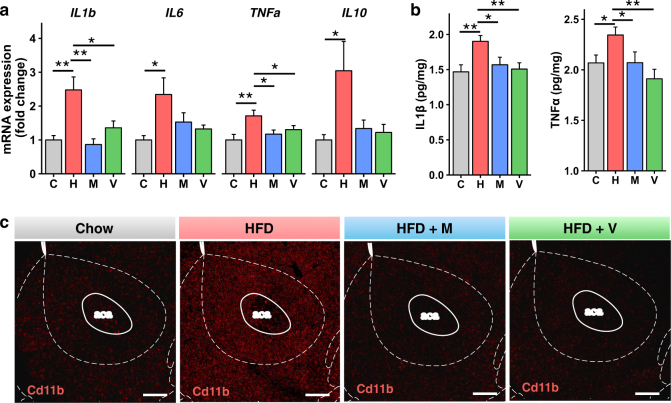
Antibiotic treatment ameliorates HFD-induced inflammation in the nucleus accumbens (Nacc). **a** mRNA abundance measured by RT qPCR in the Nacc of chow and HFD-fed mice, treated or not with antibiotics (*n* = 12/group). **b** ELISA assays for IL-1β and TNFα present in homogenates from the Nacc (*n* = 14/group). **c** Representative images of the Nacc from chow and HFD-fed mice treated or not with antibiotics, stained with Cd11b antibody (red). Scale bars, 200 μm. C chow, H HFD, M HFD + metronidazole, V HFD + vancomycin. Data are shown as mean ± SEM. *P < 0.05, **P < 0.01, and ***P < 0.001 by ANOVA, followed by pre-planned *t*-tests

Low-grade inflammation in adipose tissue and liver is an important component of the pathophysiology of insulin resistance in obesity [1, 8]. We have previously shown that treatment with vancomycin or metronidazole can decrease adipose and liver inflammation and the elevated serum tumor necrosis factor-α (TNFα) levels which occur in HFD-fed C57Bl/6J mice, and that this can be transferred to GF-mice [8]. RT-qPCR analysis of the Nacc of HFD-fed mice showed markedly increased levels of *TNFα*, *IL-1β*, *IL-6*, and *IL-10* mRNAs, and these returned to normal in HFD-fed mice treated with antibiotics (Fig. 5a). This was accompanied by similar, albeit smaller, changes in TNFα and IL-1β protein levels in the Nacc (Fig. 5b). The macrophage marker Cd11b was also increased by HFD feeding in the Nacc, and this was decreased by treatment with antibiotics (Fig. 5c and Supplementary Fig. 5a,b). Glial-fibrillary acid protein (GFAP), a marker of astrocyte activation [35], was also increased by the HFD at the protein and mRNA levels in the Nacc, but this was not reversed by antibiotic treatment (Supplementary Fig. 5c,d), while Iba1, a microglial marker, was not changed by either HFD or antibiotics (Supplementary Fig. 5a). Finally, we measured the levels of inflammatory markers in the ventral tegmental area (VTA), a region from which dopaminergic neurons project to the Nacc along the mesolimbic pathway. As in the Nacc, mRNA levels of *IL-10* and *IL-6* were increased in the VTA with the HFD and decreased to control levels by vancomycin treatment (Supplementary Fig. 5e). Overall, these findings support the hypothesis that gut microbes affect central inflammation in brain regions involved in behavioral and mood control.

### HFD and antibiotics affect the levels of metabolites and of the neurotrophic factor BDNF in the brain

**Fig. 6:**
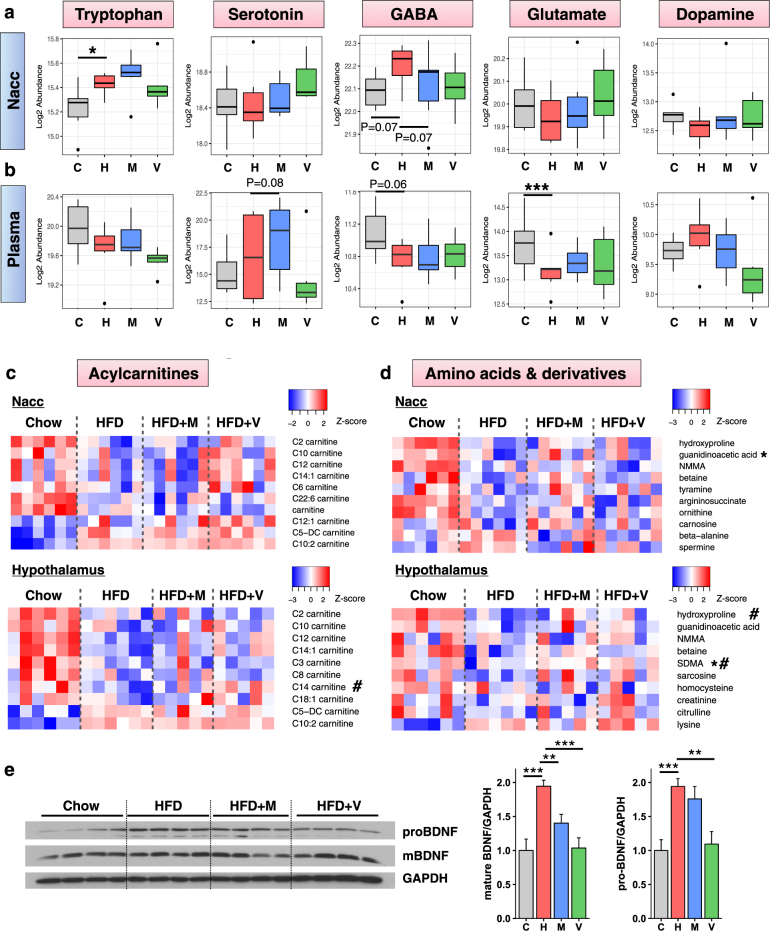
HFD affect the levels of metabolites and BDNF in the brain and in the plasma. Boxplots of neurotransmitter levels in the Nacc (**a**) and plasma (**b**) (*n* = 6/group). *P < 0.05 and **P < 0.01 by moderated *t*-tests. **c** Heat map of the top 10 acylcarnitines differentially regulated by HFD and/or antibiotics in the hypothalamus and Nacc. **d** Heat map of the top 10 amino acids and derivatives differentially regulated by HFD and/or antibiotics in the hypothalamus and Nacc. Asterisk (*) and (#) represent metabolites significantly changed by HFD compared to chow levels, and significantly reversed to control levels by metronidazole (*) or vancomycin (#). **e** Western blots and quantification of mature BDNF and pro-BDNF reported to GAPDH in the Nacc of chow and HFD-fed mice, treated or not with antibiotics (*n* = 8/group). Data are shown as mean ± SEM, normalized to chow-fed mice. **P < 0.01 and ***P < 0.001 by ANOVA, followed by pre-planned *t*-tests. C chow, H HFD, M HFD + metronidazole, V HFD + vancomycin

Microbes in the intestine produce a variety of metabolites which can be absorbed to varying degrees and further metabolized or taken up into tissues, including the brain, where they can influence metabolic, immunologic, and behavioral phenotypes [36–38]. To determine the effect of changing the gut microbiota by HFD and antibiotics on the metabolome, we performed gas chromatography coupled with mass spectrometry (GC/LC-MS) to measure the levels of 116 metabolites, including most major neurotransmitters, in plasma and in extracts from hypothalamus and Nacc. Tryptophan was significantly increased in the Nacc of HFD-fed mice, and returned toward normal in the HFD + vancomycin group (Fig. 6a). GABA followed the same trend, and tended to be decreased by metronidazole (Fig. 6a). Other neurotransmitters, such as serotonin, glutamate and dopamine, tended to be lower in HFD-fed mice and increase in HFD + vancomycin, but these changes did not reach significance (Fig. 6a). Levels of all these neurotransmitters followed similar patterns in the hypothalamus (Supplementary Fig. 6a), consistent with the strong correlation in metabolites levels between both brain regions (Supplementary Fig. 6b). These were largely independent of changes in plasma, where GABA and glutamate were decreased, and serotonin increased by HFD (Fig. 6c). Indeed, there was a poorer correlation of metabolites in plasma and brain versus the correlation between brain regions (Supplementary Fig. 6c,d).

In addition to neurotransmitters, acylcarnitines and amino acids and their derivatives were significantly changed by the HFD and by antibiotics in the brain (Fig. 6c,d). For example, C14 carnitine was significantly decreased by HFD in the hypothalamus, and this was reversed by vancomycin (Fig. 6c). By contrast, C5-DC and C10:2 carnitines both showed significant increases in Nacc and hypothalamus of HFD mice, but these did not reverse with antibiotics. Among amino acids and their derivatives, guanidinoacetic acid was significantly decreased by HFD in the Nacc, and this was reversed by metronidazole (Fig. 6d). Symmetric dimethylarginine (SDMA) and hydroxyproline were also significantly decreased by the HFD in the hypothalamus and increased toward normal by either one or both antibiotics. As for the neurotransmitters, the levels of most carnitines and amino acids were more similar between brain regions than between brain and plasma (Supplementary Fig. 6e,f).

Finally, increased brain-derived neurotrophic factor (BDNF) signaling in the Nacc has been linked to depression in humans [39], as well as greater susceptibility to the depressive effects of social defeat [39] and diet-induced obesity [20] in mice. We also observed a two-fold increase in the levels of pro and mature BDNF proteins in the Nacc after 6 weeks of HFD compared with chow-fed mice, and this was reversed with antibiotic treatment (Fig. 6e). Interestingly, this occurred with no significant change in mRNA levels of BDNF or its receptor TrkB in the Nacc (Supplementary Fig. 7a), indicating a post-transcriptional effect. In the VTA, BDNF was also significantly decreased by vancomycin treatment in HFD-fed mice (Supplementary Fig. 7b). Overall, these data show that microbial metabolites and other molecules, such as BDNF, are affected by changes in gut microbiota composition driven by HFD and antibiotics, coupled with the changes in insulin signaling and inflammation in the brain. These can contribute to the changes in metabolism and behavior.

## Discussion

Modifications in gut microbial composition induced by diet and other factors can play an important role in the metabolic changes associated with obesity, including glucose intolerance, insulin resistance and inflammation in fat and liver [1, 2, 8, 11]. These interactions are complex and depend on multiple factors, such as host genetics; diet composition and quantity; presence of probiotics, prebiotics, or antibiotics; and other environmental factors [8, 10]. In the present study, we show that modification of gut microbiota due to HFD feeding of mice results in multiple behavioral abnormalities indicative of anxiety and depression. These include increased time spent in the dark compartment of a dark-light box, increased latency to feed in a novelty-suppressed feeding test, decreased exploration in an open-field test and increased marble burying activity. We show that antibiotic treatment improves not only the metabolic abnormalities induced by the HFD, but many of the behavioral changes associated with diet-induced obesity. Indeed, two weeks of treatment with either vancomycin or metronidazole reverses HFD-induced hyperglycemia and glucose intolerance (without affecting body weight) and corrects the depressive and anxiety-like behaviors. As 6 weeks on high-fat diet led to increased body weight gain compared to chow, it is not possible to separate the effect of mild diet-induced obesity (DIO) from the effects of the high-fat diet itself on behavior. However, antibiotic treatment did not lead to changes in food intake or body composition or block the HFD-induced weight gain, although it did reverse most of the behavioral changes. Thus, whether the behavioral changes in the HFD mice are due to the diet itself or mild obesity, the response to antibiotics indicates that they are, at least in part, due to the changing microbiome. This is further supported by the fact that the effect of HFD and antibiotics improvement could be reproduced in germ-free mice by fecal transfer of gut microbiota. Mechanistically, the improved behavioral tests are associated with a reversal of brain insulin resistance and signs of brain inflammation observed in DIO mice, as well as changes in brain metabolites, some neurotransmitters and the neurotrophic factor BDNF. Although vancomycin targets gram-positive bacteria, and metronidazole targets anaerobic bacteria, in the context of HFD-induced obesity, both antibiotics have similar effects on insulin signaling, inflammation, metabolism and behavior, suggesting the gut bacteria involved are sensitive to both antibiotics.

Many clinical reports have shown that obesity and Type 2 diabetes are associated with brain disorders, including depression, anxiety, dementia, and increased rates of cognitive decline [16–18]. In mice, eating a HFD for as little as six weeks leads to neurobehavioral abnormalities, with increases in behaviors reflective of both anxiety and depression, and changing gut microbiota composition of the HFD-fed mice using antibiotics lead to recovery from the increased anxiety and depressive-like behaviors in less than two weeks, without affecting weight gain. Other studies have shown that the effects of the changing microbiome are complex and depend on many factors, including the timing and direction of change. For example, despite some limitations with the germ-free mouse model, which can also have developmental defects, these mice display decreased anxiety on behavioral testing that can be reversed by colonization with microbiota from healthy donor mice [25]. On the other hand, antibiotic treatment early in life, when the gut microbiome is developing, can induce long-term anxiety-like behaviors, as well as abnormal social behaviors in mice [40].

We have previously shown that modification of gut microbiota through antibiotic treatment in DIO mice improves insulin signaling in peripheral tissues, like muscle, fat and liver [8]. The current study shows that similar changes occur in the brain. Indeed, HFD leads to reduced phosphorylation of the IR and IRS-1 in response to exogenous insulin in multiple brain regions including the hypothalamus, which controls metabolic homeostasis, and regions of the limbic system such as the Nacc, which are known to control mood and behavior [41], consistent with other studies [34, 42]. Antibiotic treatment returns insulin stimulation to control levels. These changes in insulin signaling at the molecular level can be reproduced in germ-free mice by fecal transplantation, directly demonstrating a role of gut microbes in control of brain insulin signaling. While metronidazole is absorbed and can cross the blood-brain barrier (BBB) [43], orally administered vancomycin is not absorbed and does not cross the BBB, excluding a direct effect of antibiotics on the insulin signaling pathway [44]. Deficits in insulin signaling in the brain have been previously associated with neurobehavioral abnormalities. Indeed, anxiety and depressive-like behaviors have been observed in mice with a complete knockout of insulin signaling in the brain [28], and in mice with knockdown of insulin receptors in specific brains areas, including the hypothalamus, central amygdala, and hippocampus ([45] and manuscript in preparation). Furthermore, in humans, intranasal insulin treatment of obese men has been shown to improve mood and reduce levels of the stress hormones ACTH and cortisol [46]. Since DIO mice have both peripheral and central insulin resistance, it is possible that improved systemic insulin sensitivity and glucose homeostasis play some role in the amelioration of the behaviors of HFD-fed mice treated with antibiotics. Indeed, *db/db* obese mice treated with the insulin sensitizer rosiglitazone, which does not cross the BBB, have improved peripheral insulin sensitivity accompanied by a decrease in depressive-like behavior [47]. On the other hand, we and others have shown that brain insulin resistance can lead to peripheral metabolic abnormalities [48, 49]. Thus, it seems more likely that the decrease in insulin signaling in brain of HFD-fed mice and the improvement with antibiotics contributes to the improvements in peripheral tissue response rather than vice versa [8].

Tissue inflammation is a general feature of obesity [1, 11] and appears to play a role in the brain insulin resistance created by the changing gut microbiota. We and others have previously shown that HFD increases systemic inflammation, increasing levels of serum TNFα and macrophage infiltration in peripheral tissues that can be decreased after treatment with antibiotics [1, 8, 11]. This is mediated, at least in part, through changes in the balance between pro-inflammatory and anti-inflammatory bile acids, and changes in the bile acid receptor TGR5 [8]. Our current data show that HFD also induces inflammation in the brain, with increased levels of cytokines and Cd11b positive macrophages in the Nacc and VTA, consistent with other studies [42, 50]. As with the peripheral inflammation of HFD-fed mice, treatment with metronidazole or vancomycin decreases the levels of cytokines and the levels of Cd11b positive macrophages in the Nacc. Sampson et al. have shown that depletion of gut bacteria with antibiotics also ameliorates neuroinflammation in a mouse model of Parkinson’s disease [51]. As deleting the insulin receptor in the brain does not induce an inflammatory response, but is sufficient to induce anxiety-like behaviors [28], it seems likely that it is the brain insulin resistance, rather than HFD-induced inflammation, which acts as the causal factor in the behavioral changes, although it is likely that inflammation can make the insulin resistance worse [52].

Another possible mechanism contributing to gut-brain communication in these animals may be changes in circulating and brain metabolites. Using LCMS metabolomics, we found that tryptophan and GABA levels in the Nacc are increased by HFD feeding and that metronidazole decreases levels of GABA back to control levels. The levels of other metabolites and neurotransmitters follow distinct patterns in brain and in plasma. For example, in plasma, GABA is decreased and serotonin is increased by HFD, consistent with previous studies showing that different gut bacteria can synthesize and modify circulating levels of GABA [53] and serotonin [54]. Our data also show that in brain, levels of tryptophan, the precursor of serotonin, increase with HFD and further increase with metronidazole. These changes are opposite in direction from the changes in the blood, suggesting that increased influx of tryptophan into the brain by HFD could be related to increased blood insulin levels [55].

In addition to neurotransmitters and their precursors, a number of amino acids or their derivatives are changed in the brain by HFD and/or antibiotics. For example, guanidinoacetic acid, a precursor of creatine, is significantly reduced by HFD and increased by metronidazole in the Nacc. Guanidinoacetic acid supplementation has been shown to increase of the creatine pool in the human brain potentially restoring cellular bioenergetics in disorders characterized by low brain creatine, including neurodegenerative diseases [56]. In the periphery, guanidinoacetic acid has been shown to decrease plasma glucose, increase insulin secretion, and improve insulin sensitivity [57, 58]. On the other hand, in the hypothalamus, hydroxyproline, a modified amino acid generally related to collagen synthesis, is reduced by HFD and increased by vancomycin, with no change in blood levels. Hydroxyproline has been shown to be decreased in plasma of genetically obese mice [59] and increased in the plasma of humans and mice after treatment with metformin [60]. Rats given fish protein dietary supplements have been shown to have increased hydroxyproline In the brain, and this was associated with decreased anxiety-like behaviors [61], There are also changes in multiple acylcarnitines in brain with HFD and antibiotics. Previous studies have shown that acylcarnitines in brain are involved in stabilizing membrane composition, improving mitochondrial function, and enhancing cholinergic neurotransmission [62].

Finally, another potential mediator of the effects of the HFD on behavior is the increase in BDNF levels in the Nacc and VTA [20]. Increased BDNF in limbic regions of the brain is associated with chronic defeat stress [39], and BDNF injection into the VTA induces depressive-like behaviors in mice [63]. In our study, BDNF levels are increased in Nacc of DIO mice and normalized by antibiotic treatment. This is consistent with previous data showing that BDNF levels are lower in the cortex and hippocampus of germ-free mice compared to conventional mice [64]. BDNF is involved in synaptic vesicle maturation, and its elevation in DIO mice might cause a potentiation of synaptic transmission leading to the observed abnormalities in these mice behaviors [65].

Taken together, our data indicate that modifications in gut microbiome of DIO mice drive changes in both host metabolism and host behavior. Indeed, HFD-fed mice develop central insulin resistance and altered behaviors indicative of anxiety and depression. Antibiotic treatment of HFD-fed mice, which causes major remodeling of the gut microbiome [8], improves peripheral and central insulin sensitivity, and reverses these behavioral/mood abnormalities. These effects are reversible when antibiotics are stopped and transferable to germ-free mice by cecal bacterial transfer. These effects of gut microbiota on the brain and behavior are mediated by changes in brain insulin signaling and inflammation, and by the modulation of neurotransmitters, metabolites and other neuroactive molecules, such as BDNF. Understanding these gut-brain interactions may open novel approaches to treatment of mood and behavioral disorders.

## Electronic supplementary material

Supplemental tables and methods

Supplemental figures
