# Supplementary material for: Gut microbiota modulate neurobehavior through changes in brain insulin sensitivity and metabolism
Source: Mol Psychiatry. 2018 Jun 18;23(12):2287–301. doi: 10.1038/s41380-018-0086-5 (PMC6294739; doi:10.1038/s41380-018-0086-5)
Supplement: Supplementary file 1 — Supplemental tables and methods [file 41380_2018_86_MOESM1_ESM.docx]

**Supplementary Table 1. Primer list for quantitative PCR.**

| **Name** | **Forward sequence** | **Sequence reverse** |
| --- | --- | --- |
| BDNF | AGCTGAGCGTGTGTGACAGT | ACCCATGGGATTACACTTGG |
| Creb | CATTAACCATGCCCAATGCAG | ATGTGCGAATCTGGTATGTTT |
| CRF | CCTCAGCCGGTTCTGATCC | GCGGAAAAAGTTAGCCGCAG |
| CRH-R1 | GGGCCATTGGGAAACTTTA | ATCAGCAGGACCAGGATCA |
| CRH-R2 | TGTGGACACTTTTGGAGCAG | TGCAGTAGGTGTAGGGACCTG |
| GFAP | GAAAACCGCATCACCATTCC | CTTAATGACCTCACCATCCCG |
| IL-10 | GATTTTAATAAGCTCCAAGACCAAGGT | CTTCTATGCAGTTGATGAAGATGTCAA |
| IL-1β | GCAACTGTTCCTGAACTCAACT | ATCTTTTGGGGTCCGTCAACT |
| IL-6 | TAGTCCTTCCTACCCCAATTTCC | TTGGTCCTTAGCCACTCCTTC |
| Nos2 | CCAAGCCCTCACCTACTTCC | CTCTGAGGGCTGACACAAGG |
| POMC | GCAACCTGCTGGCTTGCATCC | CCGAAGCGGTCCCAGCGGAA |
| TBP | ACCCTTCACCAATGACTCCTATG | TGACTGCAGCAAATCGCTTGG |
| TLR4 | TTCAGAACTTCAGTGGCTGG | TGTTAGTCCAGAGAAACTTCCTG |
| TNFα | GACCCTCACACTCAGATCATCTTC | TTGCTACGACGTGGGCTAC |
| TrkB | AAGGACTTTCATCGGGAAGCTG | TCGCCCTCCACACAGACAC |

**Supplementary Table 2. MRM transitions in positive polarity used to detect the measured metabolites.**

| **Compound name** | **HMDB ID** | **Formula** | **Polarity** | **Q1** | **Q3** | **CE (V)** |
| --- | --- | --- | --- | --- | --- | --- |
| phenylalanine-d8 | Internal Standard | C9H3NO2D8 | Positive | 174.14 | 128.10 | 17 |
| valine-d8 | Internal Standard | C5H3NO2D8 | Positive | 126.14 | 80.20 | 9 |
| 1-methylhistamine | HMDB00898 | C6H11N3 | Positive | 126.11 | 109 | 13 |
| 2-deoxycytidine | HMDB00014 | C9H13N3O4 | Positive | 228.10 | 112.00 | 9 |
| 3-indoleacetic acid | HMDB00197 | C10H9NO2 | Positive | 176.07 | 130.10 | 25 |
| 5-adenosylhomocysteine | HMDB00939 | C14H20N6O5S | Positive | 385.13 | 136.10 | 21 |
| 5-HIAA | HMDB00763 | C10H9NO3 | Positive | 192.07 | 146.00 | 21 |
| 5-hydroxytryptophan | HMDB00472 | C11H12N2O3 | Positive | 221.09 | 204.20 | 9 |
| 5-hydroxytryptophol | HMDB01855 | C10H11NO2 | Positive | 178.09 | 160.10 | 13 |
| 5-methoxytryptophol | HMDB01896 | C11H13NO2 | Positive | 192.10 | 174.10 | 13 |
| acetylcholine | HMDB00895 | C7H16NO2 | Positive | 147.13 | 43.30 | 37 |
| adenine | HMDB00034 | C5H5N5 | Positive | 136.06 | 119.00 | 24 |
| adenosine | HMDB00050 | C10H13N5O4 | Positive | 268.11 | 136.00 | 17 |
| ADMA | HMDB01539 | C8H18N4O2 | Positive | 203.15 | 70.20 | 33 |
| alanine | HMDB00161 | C3H7NO2 | Positive | 90.06 | 44.30 | 13 |
| alpha-glycerophosphocholine | HMDB00086 | C8H20NO6P | Positive | 258.11 | 104.10 | 17 |
| anthranilic acid | HMDB01123 | C7H7NO2 | Positive | 138.06 | 120.00 | 9 |
| argininosuccinate | HMDB00052 | C10H18N4O6 | Positive | 291.13 | 70.20 | 35 |
| asparagine | HMDB00168 | C4H8N2O3 | Positive | 133.06 | 74.10 | 17 |
| aspartate | HMDB00191 | C4H7NO4 | Positive | 134.05 | 74.10 | 17 |
| beta-alanine | HMDB00056 | C3H7NO2 | Positive | 90.06 | 72.10 | 5 |
| betaine | HMDB00043 | C5H11NO2 | Positive | 118.09 | 58.20 | 33 |
| butyrobetaine | HMDB01161 | C7H16NO2 | Positive | 147.13 | 88.10 | 17 |
| C10 carnitine | HMDB00651 | C17H33NO4 | Positive | 316.25 | 85.10 | 31 |
| C10:2 carnitine | HMDB13325 | C17H29NO4 | Positive | 312.60 | 85.40 | 20 |
| C12 carnitine | HMDB02250 | C19H37NO4 | Positive | 344.60 | 85.10 | 20 |
| C12:1 carnitine | HMDB13326 | C19H35NO4 | Positive | 342.60 | 85.10 | 20 |
| C14 carnitine | HMDB05066 | C21H41NO4 | Positive | 372.31 | 85.10 | 27 |
| C14:1 carnitine | HMDB02014 | C21H39NO4 | Positive | 370.50 | 85.20 | 20 |
| C14:2 carnitine | HMDB13331 | C21H37NO4 | Positive | 368.50 | 85.20 | 20 |
| C16 carnitine | HMDB00222 | C23H45NO4 | Positive | 400.34 | 85.10 | 35 |
| C16-OH carnitine | HMDB13337 | C23H45NO5 | Positive | 416.70 | 85.10 | 20 |
| C18 carnitine | HMDB00848 | C25H49NO4 | Positive | 428.70 | 85.10 | 20 |
| C18:1-OH carnitine | HMDB13339 | C25H47NO5 | Positive | 442.70 | 85.10 | 20 |
| C18:2 carnitine | HMDB06469 | C25H45NO4 | Positive | 424.70 | 85.10 | 20 |
| C2 carnitine | HMDB00201 | C9H17NO4 | Positive | 204.13 | 85.10 | 19 |
| C20 carnitine | HMDB06460 | C27H53NO4 | Positive | 456.70 | 85.10 | 20 |
| C20:4 carnitine | N/A | C27H45NO4 | Positive | 448.70 | 85.10 | 20 |
| C22:6 carnitine | HMDB06510 | C29H45NO4 | Positive | 472.60 | 85.10 | 20 |
| C3 carnitine | HMDB00824 | C10H19NO4 | Positive | 218.40 | 85.10 | 20 |
| C3-DC carnitine | HMDB02095 | C10H17NO6 | Positive | 248.40 | 85.10 | 20 |
| C3-DC-CH3 carnitine | HMDB13133 | C11H19NO6 | Positive | 262.40 | 85.10 | 20 |
| C4 carnitine | HMDB02013 | C11H21NO4 | Positive | 232.40 | 85.10 | 20 |
| C4-OH carnitine | HMDB13127 | C11H21NO5 | Positive | 248.40 | 85.10 | 20 |
| C5 carnitine | HMDB00688 | C12H23NO4 | Positive | 246.50 | 85.10 | 20 |
| C5:1 carnitine | HMDB02366 | C12H21NO4 | Positive | 244.50 | 85.10 | 20 |
| C5-DC carnitine | HMDB13130 | C12H21NO6 | Positive | 276.50 | 85.10 | 20 |
| C6 carnitine | HMDB00705 | C13H25NO4 | Positive | 260.19 | 85.00 | 19 |
| C7 carnitine | HMDB13238 | C14H27NO4 | Positive | 274.50 | 85.10 | 20 |
| C8 carnitine | HMDB00791 | C15H29NO4 | Positive | 288.22 | 85.10 | 23 |
| C9 carnitine | HMDB13288 | C16H31NO4 | Positive | 302.50 | 85.10 | 20 |
| cAMP | HMDB00058 | C10H12N5O6P | Positive | 330.06 | 136.20 | 25 |
| carnitine | HMDB00062 | C7H15NO3 | Positive | 162.12 | 85.10 | 21 |
| carnosine | HMDB00033 | C9H14N4O3 | Positive | 227.12 | 110.10 | 25 |
| choline | HMDB00097 | C5H14NO | Positive | 105.12 | 61.30 | 17 |
| citrulline | HMDB00904 | C6H13N3O3 | Positive | 176.11 | 70.10 | 25 |
| cotinine | HMDB01046 | C10H12N2O | Positive | 177.10 | 80.10 | 25 |
| creatine | HMDB00064 | C4H9N3O2 | Positive | 132.08 | 90.10 | 9 |
| creatinine | HMDB00562 | C4H7N3O | Positive | 114.07 | 44.30 | 17 |
| cytosine | HMDB00630 | C4H5N3O | Positive | 112.05 | 94.90 | 21 |
| dimethylglycine | HMDB00092 | C4H9NO2 | Positive | 104.07 | 58.20 | 13 |
| DOPA | HMDB00181 | C9H11NO4 | Positive | 198.08 | 152.10 | 13 |
| dopamine | HMDB00073 | C8H11NO2 | Positive | 154.09 | 91.10 | 29 |
| epinephrine | HMDB00068 | C9H13NO3 | Positive | 184.10 | 166.00 | 5 |
| GABA | HMDB00112 | C4H9NO2 | Positive | 104.07 | 87.10 | 9 |
| glutamate | HMDB00148 | C5H9NO4 | Positive | 148.06 | 84.00 | 17 |
| glycine | HMDB00123 | C2H5NO2 | Positive | 76.04 | 30.40 | 9 |
| guanidinoacetic acid | HMDB00128 | C3H7N3O2 | Positive | 118.06 | 30.40 | 21 |
| guanine | HMDB00132 | C5H5N5O | Positive | 152.06 | 135.00 | 20 |
| histidine | HMDB00177 | C6H9N3O2 | Positive | 156.08 | 110.00 | 13 |
| homocysteine | HMDB00742 | C4H9NO2S | Positive | 136.05 | 90.00 | 9 |
| hydroxyproline | HMDB00725 | C5H9NO3 | Positive | 132.07 | 86.10 | 13 |
| hypoxanthine | HMDB00157 | C5H4N4O | Positive | 137.05 | 119.00 | 24 |
| isoleucine | HMDB00172 | C6H13NO2 | Positive | 132.10 | 86.10 | 9 |
| kynurenic acid | HMDB00715 | C10H7NO3 | Positive | 190.05 | 144.10 | 17 |
| leucine | HMDB00687 | C6H13NO2 | Positive | 132.10 | 86.10 | 9 |
| kynurenine | HMDB00684 | C10H12N2O3 | Positive | 209.09 | 94.00 | 13 |
| tryptophan | HMDB00929 | C11H12N2O2 | Positive | 205.10 | 187.90 | 9 |
| lysine | HMDB00182 | C6H14N2O2 | Positive | 147.12 | 84.20 | 21 |
| methionine | HMDB00696 | C5H11NO2S | Positive | 150.06 | 61.10 | 21 |
| methionine sulfoxide | HMDB02005 | C5H11NO3S | Positive | 166.06 | 74.10 | 13 |
| methylthioadenosine | HMDB01173 | C11H15N5O3S | Positive | 298.10 | 136.00 | 17 |
| mevalonic acid | HMDB00227 | C6H12O4 | Positive | 149.08 | 65.10 | 29 |
| N-acetylserotonin | HMDB01238 | C12H14N2O2 | Positive | 219.12 | 160.00 | 9 |
| N-carbamoyl-beta-alanine | HMDB00026 | C4H8N2O3 | Positive | 133.06 | 115.00 | 5 |
| niacinamide | HMDB01406 | C6H6N2O | Positive | 123.06 | 80.10 | 25 |
| N-methyltryptamine | HMDB04370 | C11H14N2 | Positive | 175.13 | 144.10 | 13 |
| NMMA | HMDB29416 | C7H16N4O2 | Positive | 189.14 | 70.20 | 29 |
| ornithine | HMDB00214 | C5H12N2O2 | Positive | 133.10 | 70.20 | 21 |
| phenylalanine | HMDB00159 | C9H11NO2 | Positive | 166.09 | 120.10 | 13 |
| phosphocholine | HMDB01565 | C5H15NO4P | Positive | 185.08 | 87.10 | 17 |
| phosphoethanolamine | HMDB00224 | C2H8NO4P | Positive | 142.03 | 44.30 | 9 |
| pipecolic acid | HMDB00716 | C6H11NO2 | Positive | 130.09 | 84.10 | 17 |
| picolinic acid | HMDB02243 | C6H5NO2 | Positive | 124.00 | 78.00 | 17 |
| proline | HMDB00162 | C5H9NO2 | Positive | 116.07 | 70.10 | 21 |
| putrescine | HMDB01414 | C4H12N2 | Positive | 89.11 | 72.20 | 9 |
| quinolinic acid | HMDB00232 | C7H5NO4 | Positive | 168.03 | 149.90 | 9 |
| sarcosine | HMDB00271 | C3H7NO2 | Positive | 90.06 | 44.30 | 11 |
| SDMA | HMDB03334 | C8H18N4O2 | Positive | 203.10 | 70.30 | 33 |
| serine | HMDB00187 | C3H7NO3 | Positive | 106.05 | 60.20 | 11 |
| serotonin | HMDB00259 | C10H12N2O | Positive | 177.10 | 160.00 | 5 |
| spermidine | HMDB01257 | C7H19N3 | Positive | 146.17 | 72.10 | 15 |
| spermine | HMDB01256 | C10H26N4 | Positive | 202.22 | 112.10 | 19 |
| taurine | HMDB00251 | C2H7NO3S | Positive | 126.02 | 44.30 | 23 |
| thiamine | HMDB00235 | C12H17N4OS | Positive | 266.12 | 122.10 | 15 |
| thymidine | HMDB00273 | C10H14N2O5 | Positive | 243.10 | 127.00 | 11 |
| thymine | HMDB00262 | C5H6N2O2 | Positive | 127.10 | 110.00 | 16 |
| thyroxine | HMDB00248 | C15H11I4NO4 | Positive | 777.70 | 731.50 | 23 |
| triiodothyronine | HMDB00265 | C15H12I3NO4 | Positive | 651.80 | 605.60 | 27 |
| trimethylamine-N-oxide | HMDB00925 | C3H9NO | Positive | 76.08 | 58.20 | 23 |
| tryptamine | HMDB00303 | C10H12N2 | Positive | 161.10 | 144.10 | 5 |
| tyramine | HMDB00306 | C8H11NO | Positive | 138.09 | 121.00 | 11 |
| tyrosine | HMDB00158 | C9H11NO3 | Positive | 182.08 | 136.10 | 11 |
| valine | HMDB00883 | C5H11NO2 | Positive | 118.09 | 72.20 | 11 |
| xanthosine | HMDB00299 | C10H12N4O6 | Positive | 285.09 | 153.10 | 11 |

**Supplementary Methods**

**Metabolomic analysis.**

**Sample processing.** Frozen brain tissue samples were homogenized in 4 volumes of HPLC water (J. T. Baker) using a TissueLyser II (Qiagen) with 3mm tungsten beads at 20 Hz in two 2-min cycles. Homogenates were then aliquoted for profiling.

**Data acquisition.** Polar metabolites were profiled using liquid chromatography tandem mass spectrometry (LC-MS). Positive ionization mode data were acquired using a 6495 triple quadrupole mass spectrometer coupled to a 1290 Infinity II U-HPLC system (Agilent). Plasma or brain homogenates (10 µL) were extracted using 90 µL of 74.9:24.9:0.2 (v/v/v) acetonitrile/methanol/formic acid containing stable isotope-labeled internal standards (0.2 ng/µL valine-d8, Isotec; and 0.2 ng/µL phenylalanine-d8 (Cambridge Isotope Laboratories)). The samples were centrifuged (10 min, 9000 g, 4ºC) and the supernatants (10 µL) were injected onto a 150 x 2.1 mm Atlantis HILIC column (Waters). The column was eluted isocratically at a flow rate of 250 µL/min with 5% mobile phase A (10 mM ammonium formate and 0.1% formic acid in water) for 1 min followed by a linear gradient to 40% mobile phase B (acetonitrile with 0.1% formic acid) over 10 min. MS data were acquired using multiple reaction monitoring and retention times, mass transitions, and collision energies were determined using authentic reference standards **(Supplementary Table 2)**. Other MS parameters were: ion spray voltage, 3.0 kV; source temperature, 200°C; nozzle voltage, 500 V; gas flow, 14 L/min; nebulizer, 40 psi; sheath gas, 250°C; sheath gas flow, 1 L/min; iFunnel high pressure RF, 90; and low pressure RF, 90. Raw data were processed using MassHunter software (Agilent) for automated peak integration. Metabolite peaks were manually reviewed for quality of integration and compared against standard reference standards to confirm identities.
