# Supplementary material for: Gut microbiota modulate neurobehavior through changes in brain insulin sensitivity and metabolism
Source: Mol Psychiatry. 2018 Jun 18;23(12):2287–301. doi: 10.1038/s41380-018-0086-5 (PMC6294739; doi:10.1038/s41380-018-0086-5)
Supplement: Supplementary file 2 — Supplemental figures [file 41380_2018_86_MOESM2_ESM.pdf]

Supplementary Figure 1

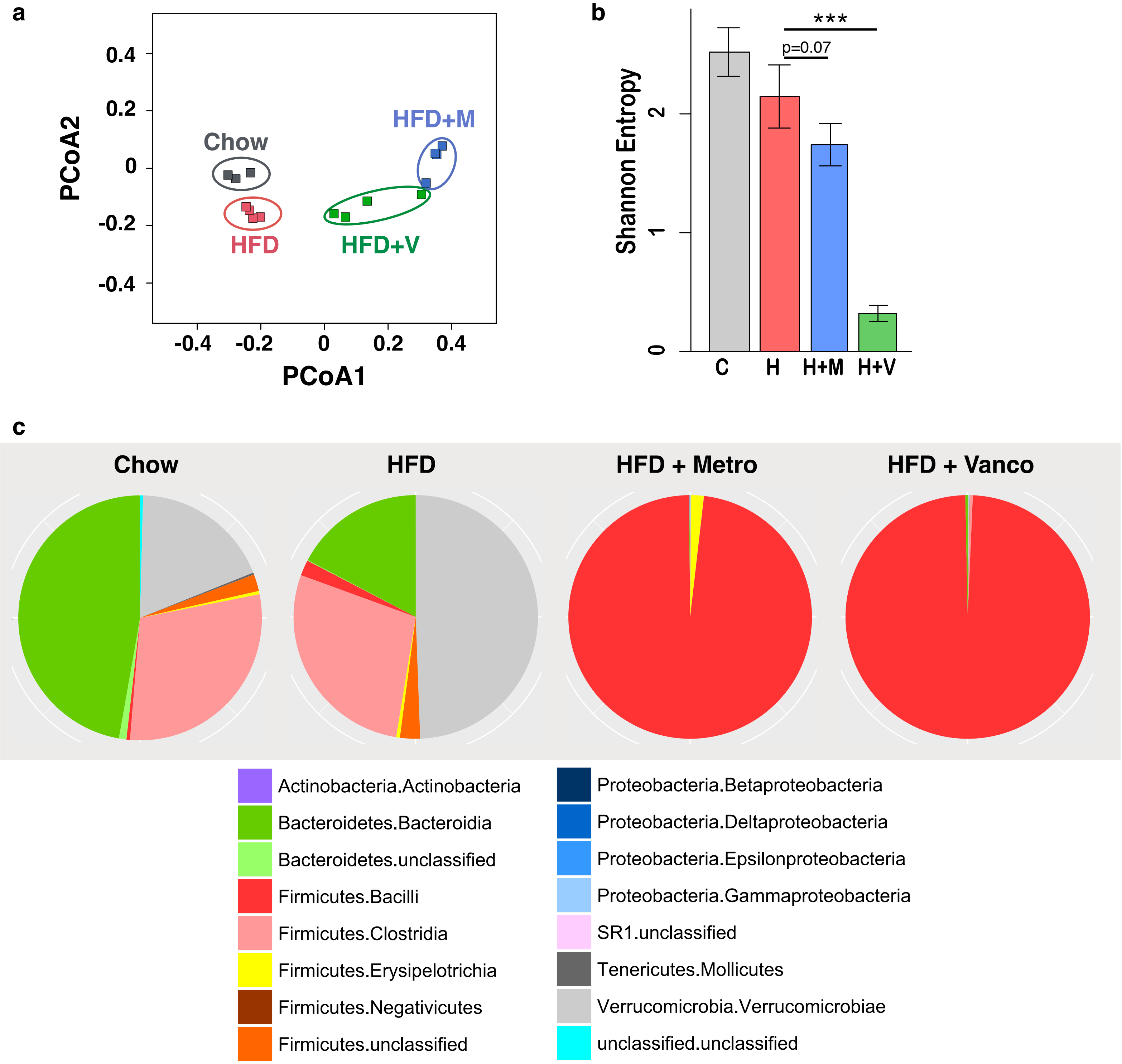

Supplementary Figure 1. Effect of HFD and antibiotic treatment on the gut microbial communities. (a) Principal component analysis (PCoA) of cecal 16S rRNA sequencing data for B6J after 4 weeks on chow or HFD and 5 weeks on placebo or either antibiotic. **(b)** Shannon entropy for gut microbial communities of mice fed chow, HFD, or HFD + antibiotics (n=3-4/group). ***P < 0.001 by ANOVA, followed by pre-planned t-tests. **(c)** Representation of bacterial class in cecal samples of mice from each group (n=3-4). C: chow, H: HFD, M: HFD+metronidazole, V: HFD+vancomycin.

Supplementary Figure 2

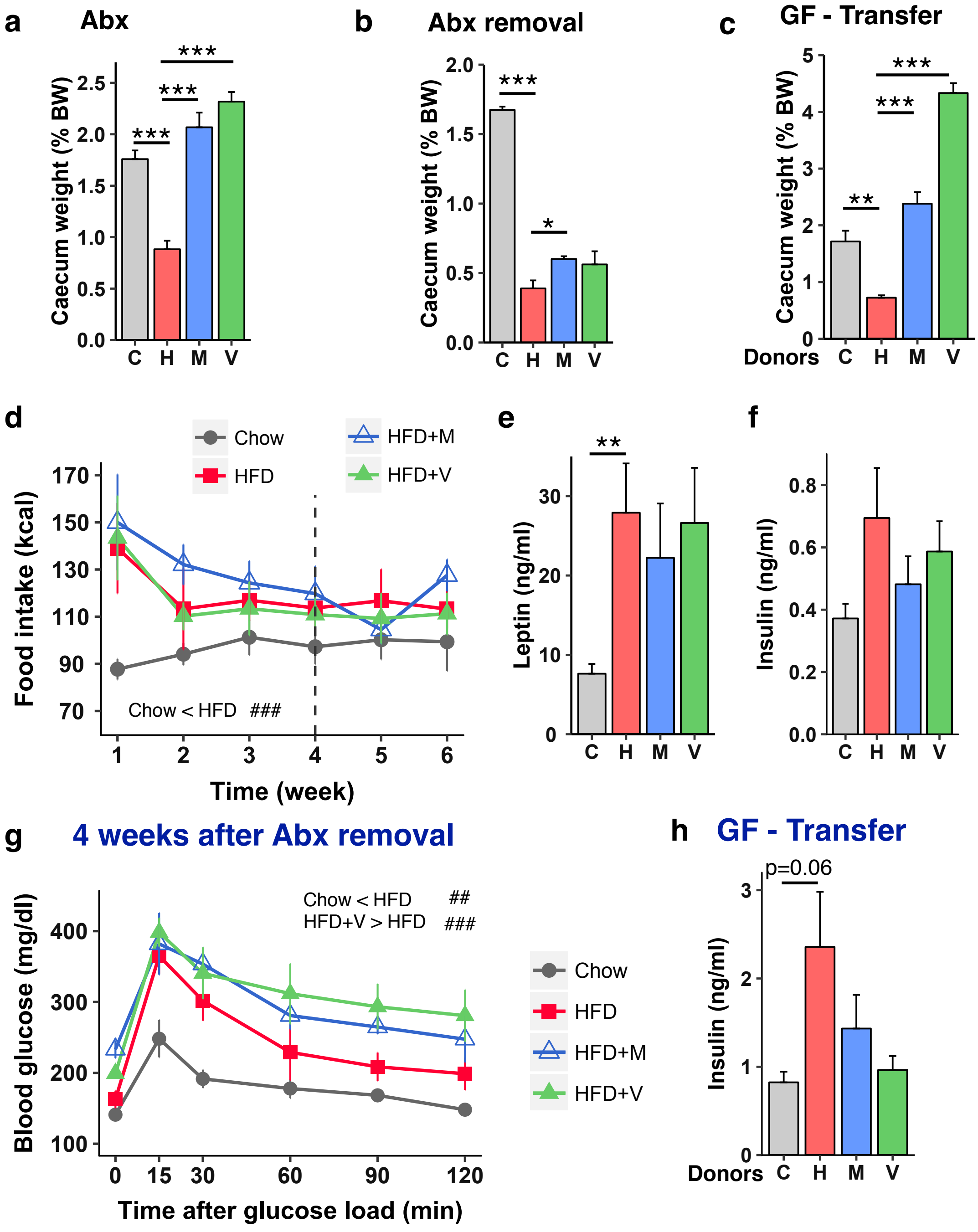

Supplementary Figure 2. Effect of antibiotic treatment on metabolism in HFD-fed mice. (a-c) Caecum weight of mice fed chow, HFD, or HFD+Abx (n=10/group) **(a)**, of mice fed chow, HFD or HFD+Abx for 2 weeks, then switched to placebo (n=4/group) **(b),** and of chow or HFD-fed GF mice colonized with donors fed chow, HFD, or HFD+Abx (n=6/group) **(c). (d)** Food intake of mice treated for 6 weeks with chow, HFD, or HFD+Abx (metronidazole (HFD+M) or vancomycin (HFD+V)) (n=7/group, dashed line = beginning of Abx treatment). **(e,f)** Leptin and insulin levels of mice fed chow, HFD, or HFD+Abx (n=10/group). **(g)** OGTT of mice fed chow, HFD or HFD+Abx for 2 weeks, then switched to placebo (n=4/group). **(h)** Insulin levels of chow or HFD recipient GF mice after bacterial transfer from mice fed chow, HFD, or HFD+Abx (n=6/group). Abx: antibiotics, C: chow, H: HFD, M: HFD+metronidazole, V: HFD+vancomycin. ## $P < 0.01$ and ### $P < 0.001$ by repeated-measure ANOVA. *$P < 0.05$, **$P < 0.01$, and ***$P < 0.001$ by ANOVA, followed by pre-planned t-tests.

Supplementary Figure 3

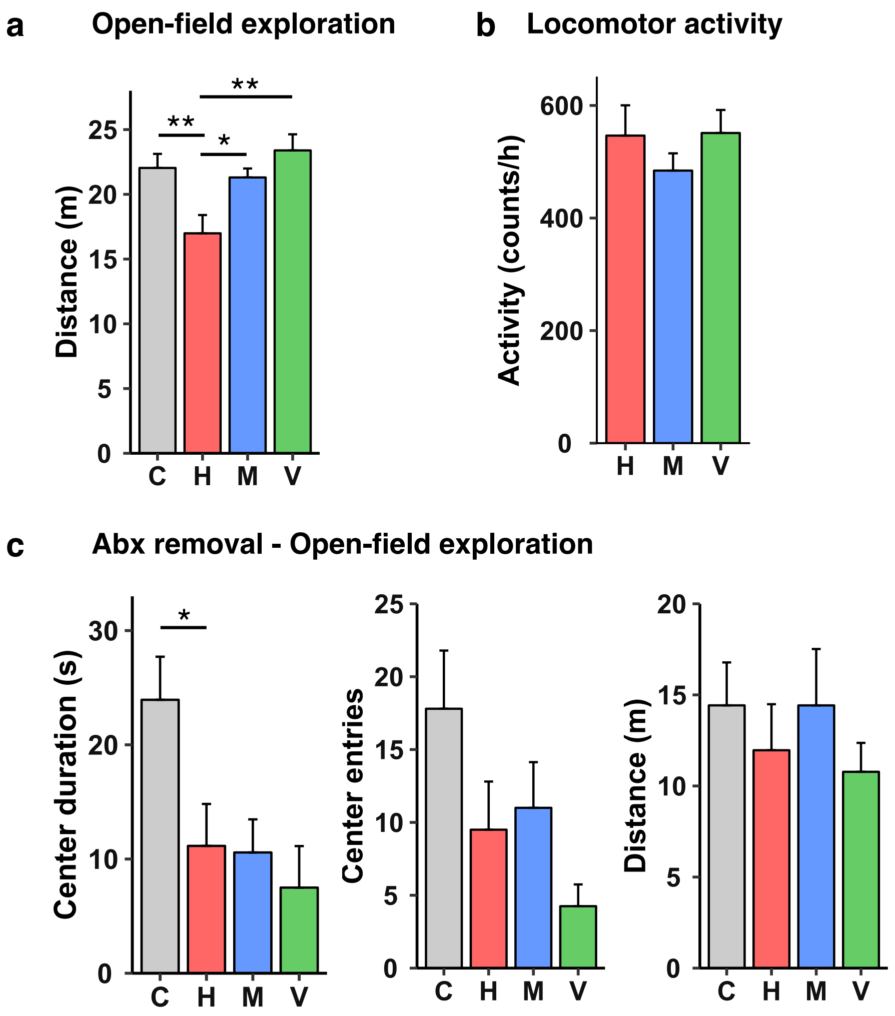

Supplementary Figure 3. Antibiotic treatment reverses HFD-induced depressive and anxiety-like behaviors. (a) Distance covered during open-field exploration of mice fed chow, HFD, or HFD+Abx (n=18/group). **(b)** Locomotor activity measured in metabolic cages of mice fed HFD or HFD+Abx (n=16/group). **(c)** Time spent in the central zone, number of entries in that area, and distance covered during open-field exploration of mice fed chow, HFD or HFD+Abx for 2 weeks, then switched to placebo (n=4-5/group). C: chow, H: HFD, M: HFD+metronidazole, V: HFD+vancomycin. Data are shown as mean ± SEM. *P < 0.05, **P < 0.01, and ***P < 0.001 by ANOVA, followed by pre-planned t-tests.

Supplementary Figure 4

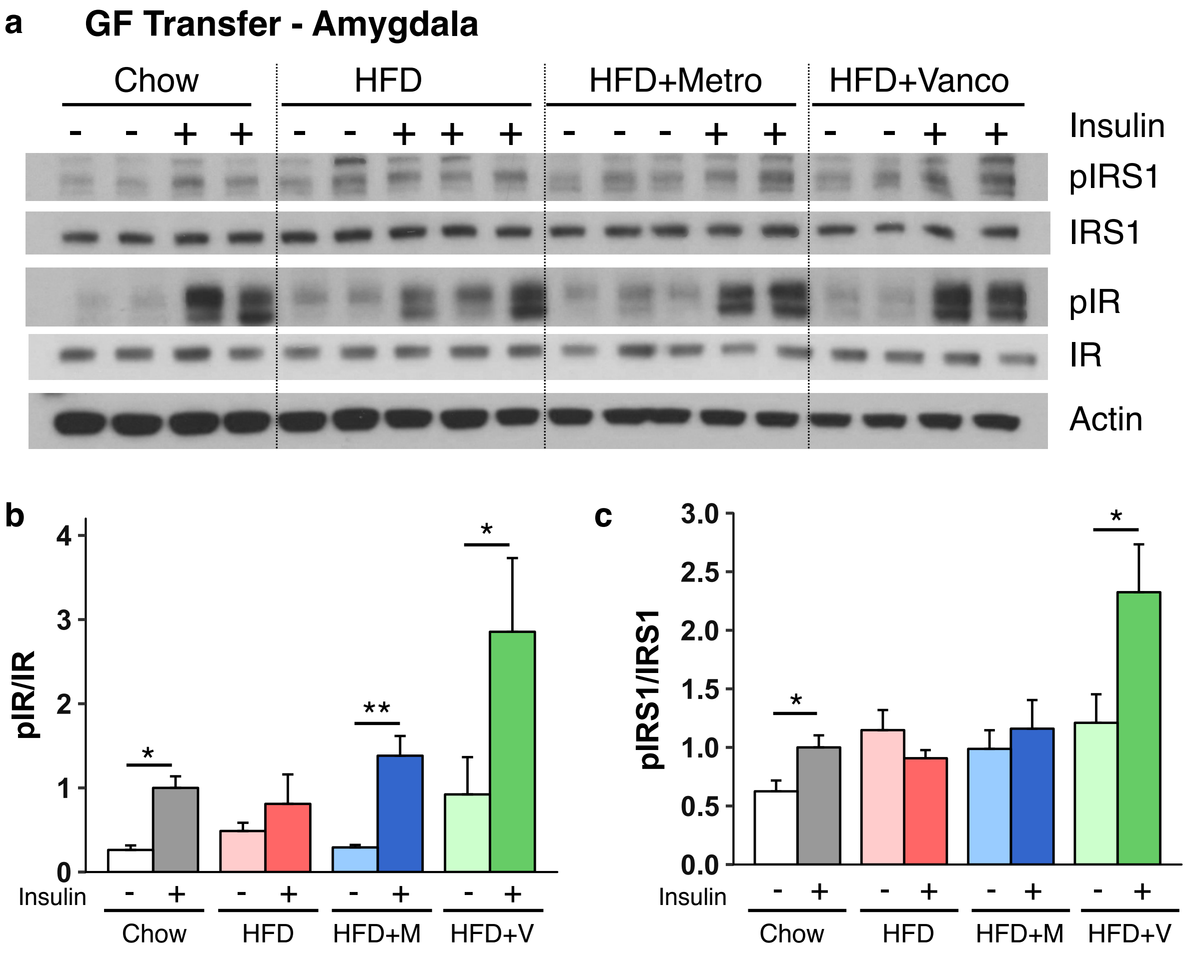

Supplementary Figure 4. Gut microbiota directly modulates brain insulin signaling. (a) Representative western blots of insulin signaling in amygdala extracts from GF mice colonized with gut microbiota from mice fed chow, HFD or HFD+Abx, after vena cava injection of saline or 5 U insulin. Actin served as a loading control. **(b,c)** Quantification of IR and IRS1 phosphorylation (pIR and pIRS1) normalized by total protein levels in the amygdala (n=4/group). M: metronidazole, V: vancomycin. Data are shown as mean ± SEM. *$P < 0.05$ and **$P < 0.01$ by pre-planned t-tests.

Supplementary Figure 5

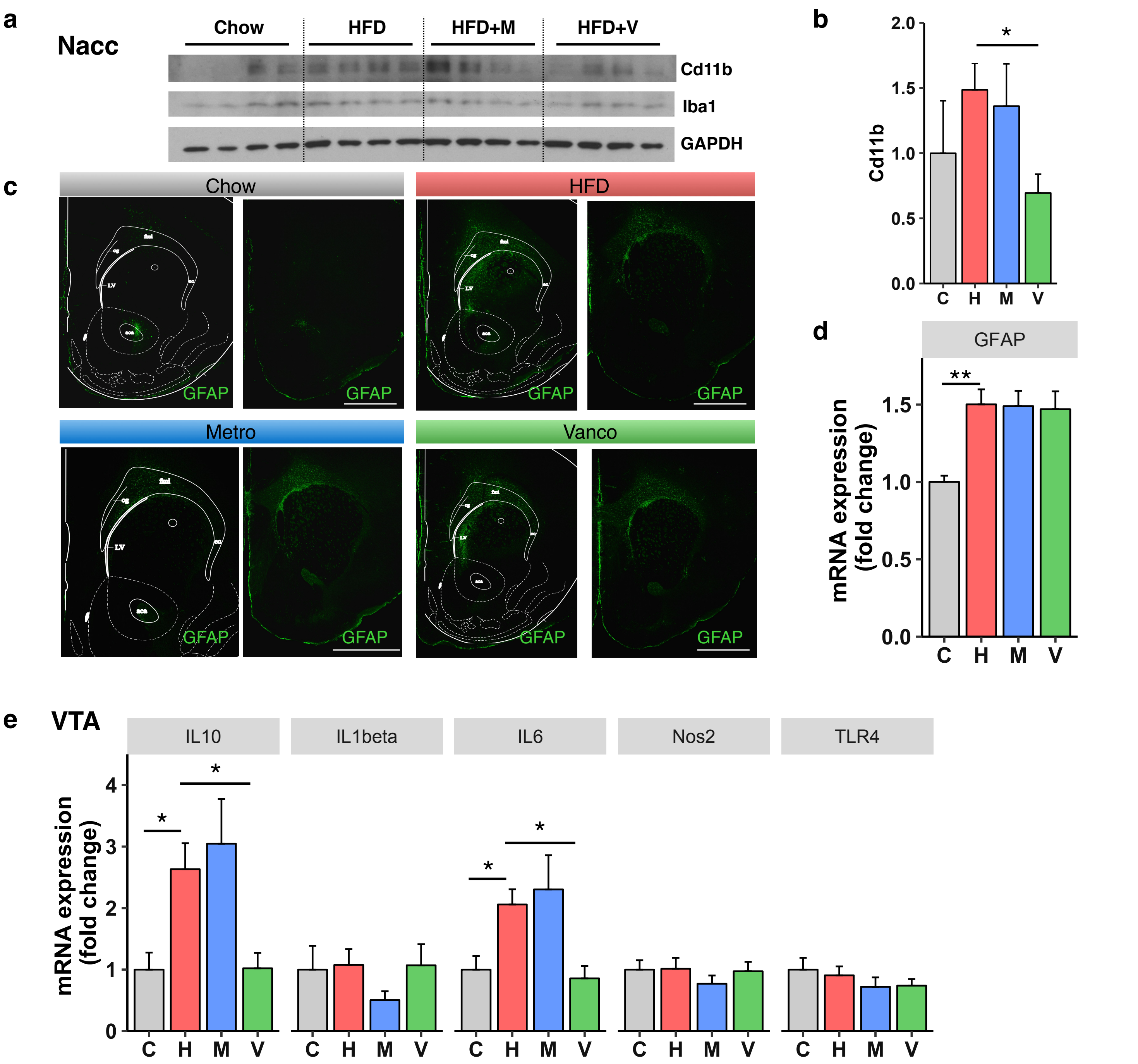

Supplementary Figure 5. Antibiotic treatment ameliorates HFD-induced inflammation in the brain. (a,b) Representative western blot of Cd11b and Iba1 **(a)** and quantification of Cd11b protein normalized to GAPDH (n=4/group) **(b)** in the Nacc of mice fed chow, HFD and HFD+Abx. **(c,d)** Representative images of GFAP staining (scale bars, 1 mm) **(c)** and *GFAP* mRNA expression (n=6/group) **(d)** in the Nacc of mice fed chow, HFD and HFD+Abx. **(e)** mRNA abundance in the VTA of mice fed chow, HFD and HFD+Abx (n=6/group). Abx: Antibiotics, C: chow, H: HFD, M: HFD+metronidazole, V: HFD+vancomycin. Data are shown as mean ± SEM. $^*P < 0.05$, $^{**}P < 0.01$, and $^{***}P < 0.001$ by ANOVA, followed by pre-planned t-tests.

Supplementary Figure 6

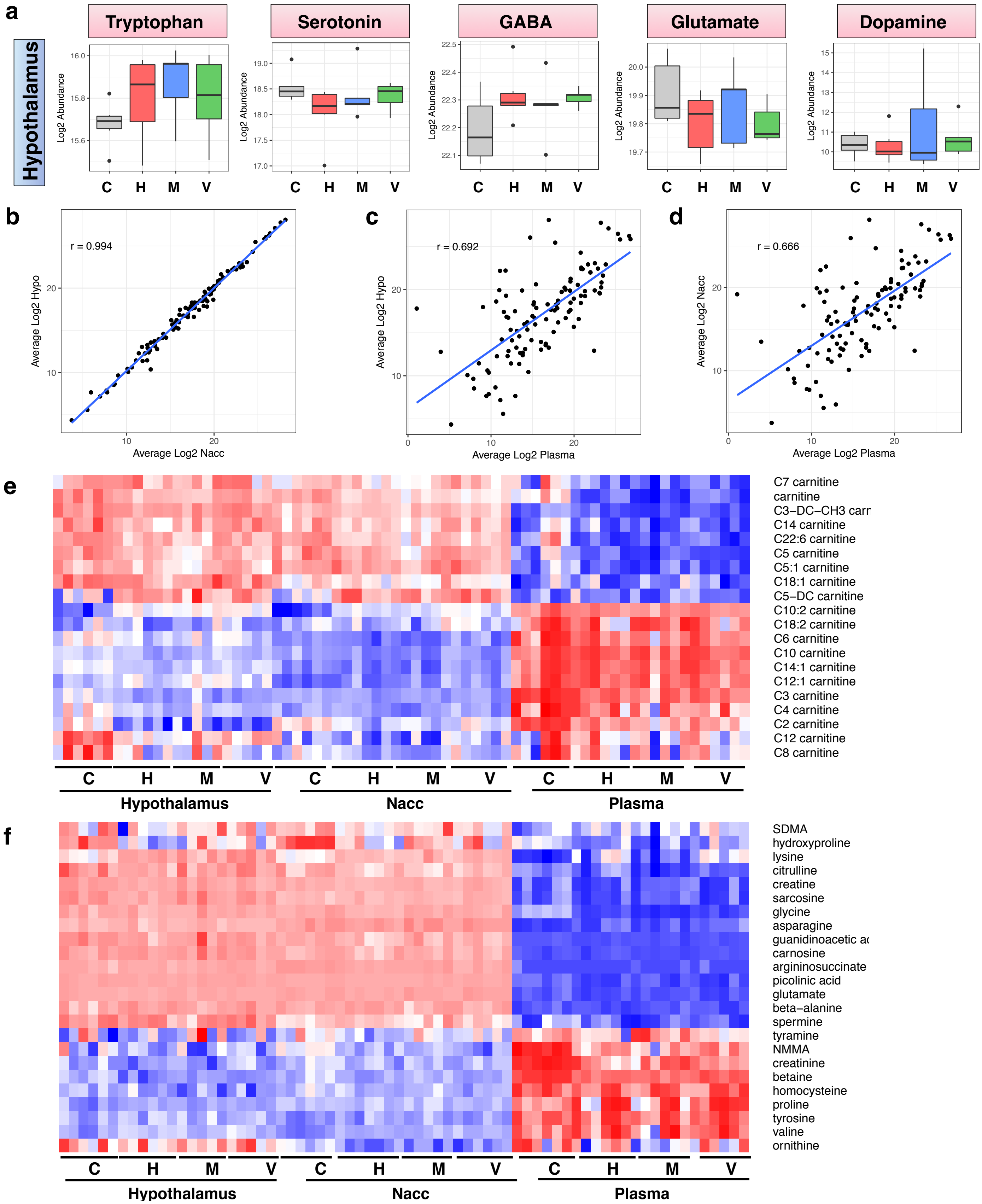

Supplementary Figure 6. Diet and antibiotics influence metabolites differently in the in brain and plasma. (a) Boxplots of neurotransmitter levels in the hypothalamus. **(b,c,d)** Correlations of log fold change between hypothalamic and Nacc contents **(b)** and between brain regions and plasma **(c,d)** in chow-fed mice. **(e,f)** Heat map of the top carnitines and amino acids that are differentially regulated by HFD, antibiotics and by tissue.

Supplementary Figure 7

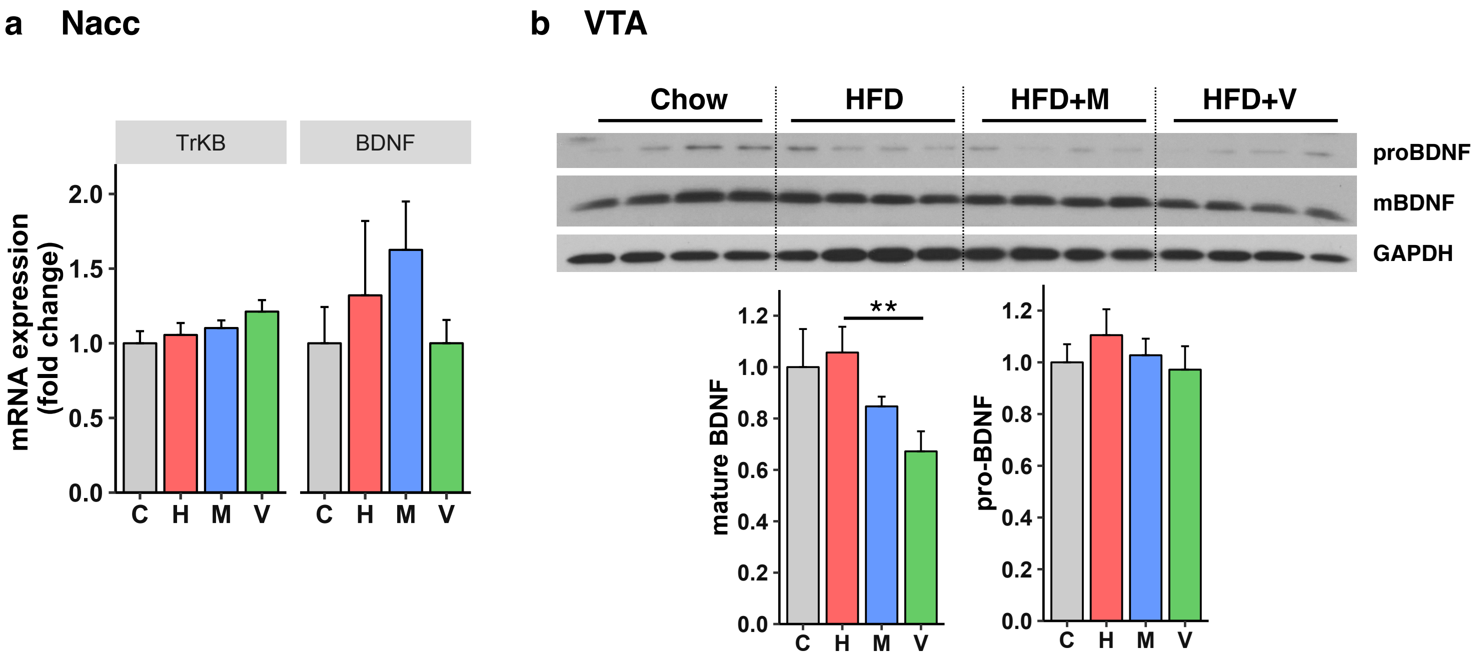

Supplementary Figure 7. Antibiotic treatment ameliorates BDNF signaling in HFD-fed mice. **(a)** mRNA abundance measured by real-time qPCR in the Nacc of mice fed chow, HFD and HFD+Abx (n=6/group). **(b)** Western blots and quantification of mature BDNF and pro-BDNF normalized to GAPDH in the ventral tegmental area (VTA) of mice fed chow, HFD and HFD+Abx (n=8/group). C: chow, H: HFD, M: HFD+metronidazole, V: HFD+vancomycin. Data are shown as mean ± SEM.**P < 0.01 by ANOVA, followed by pre-planned t-tests.

Supplementary Figure 8

Figure 3a Hypothalamus

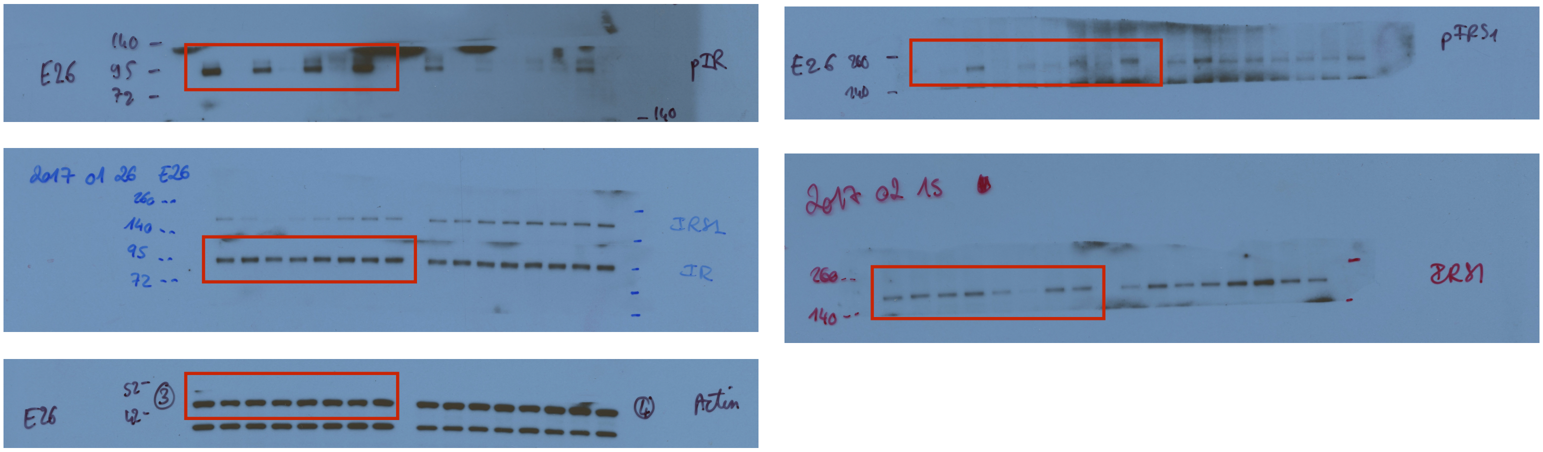

Figure 3d Nacc

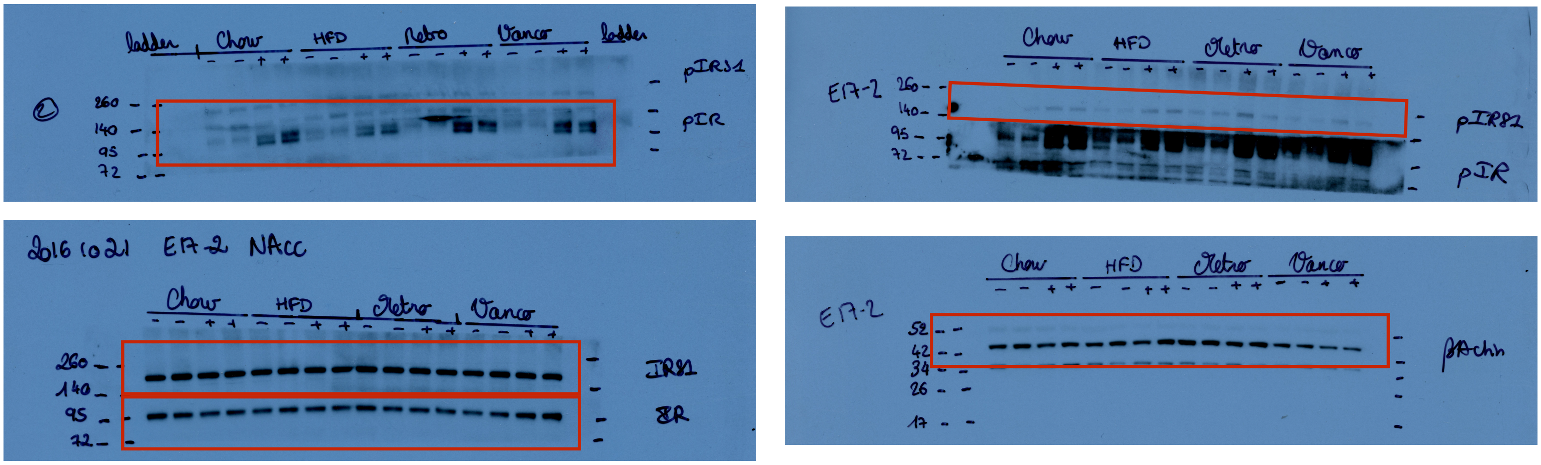

Figure 4b GF Hypothalamus

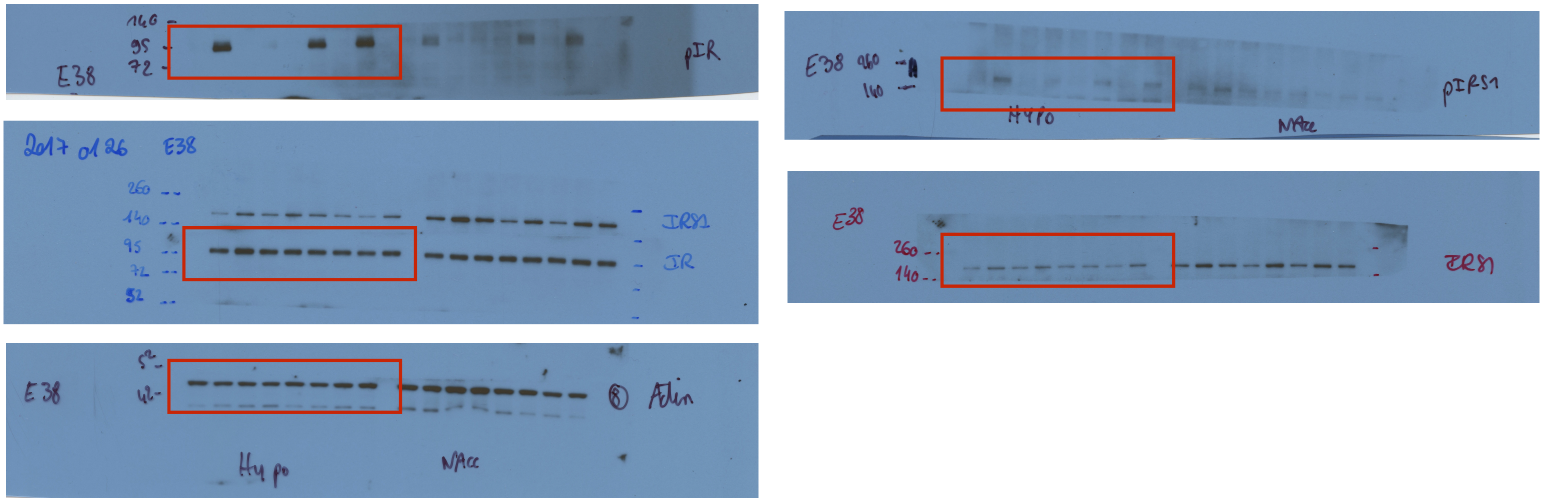

Supplementary Figure 8. Uncut blots. The red sections indicate blot results shown in the indicated figures.

Supplementary Figure 9

Figure 4e GF Nacc

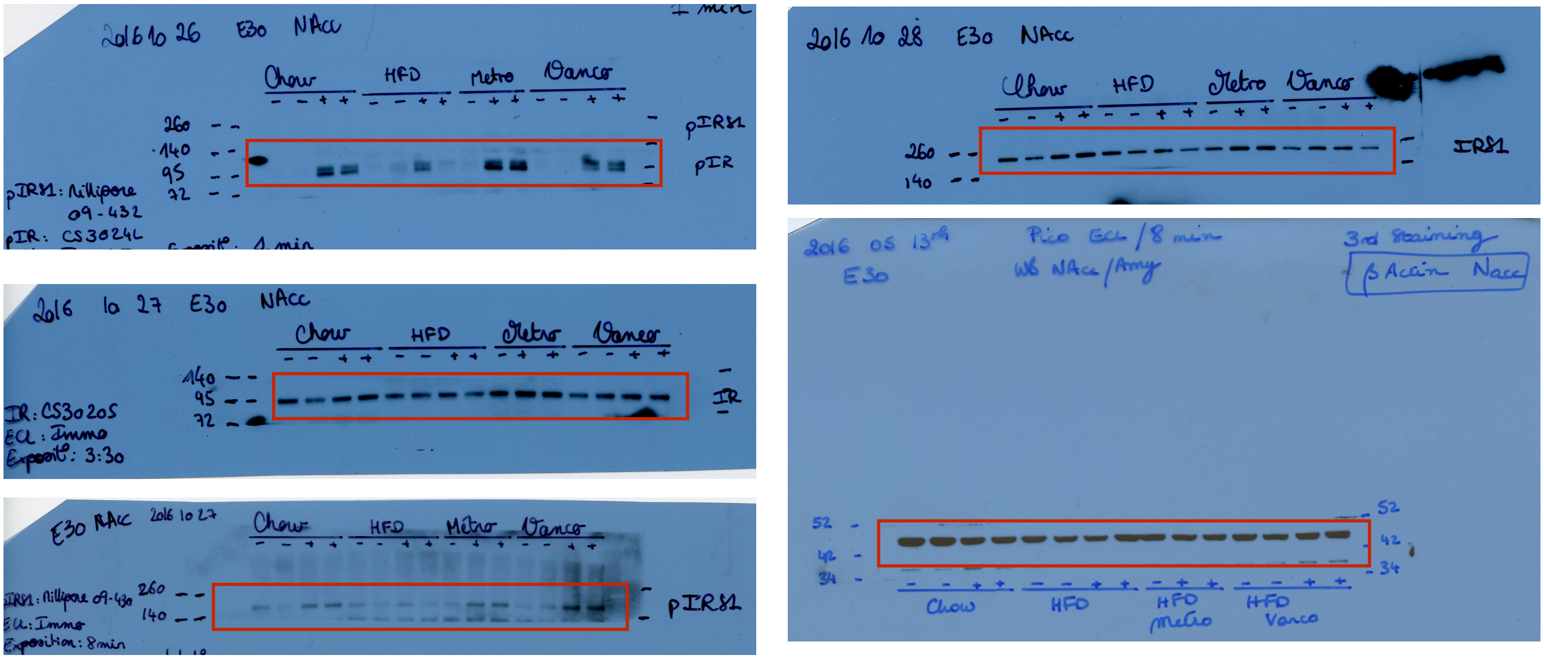

Figure 6e BDNF

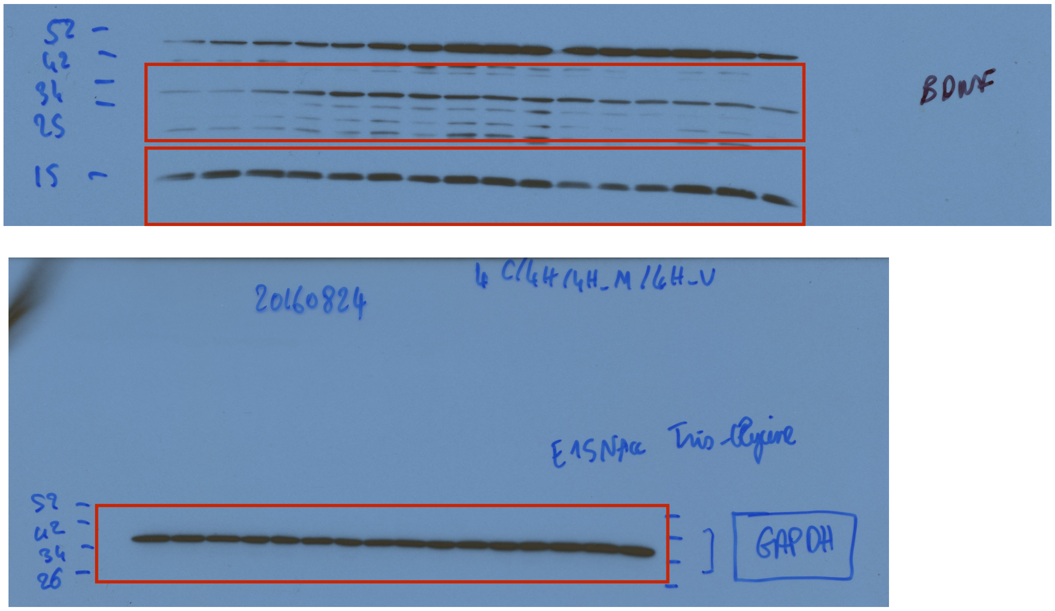

Supplementary Figure 9. Uncut blots. The red sections indicate blot results shown in the indicated figures.

Supplementary Figure 10

Figure S4a

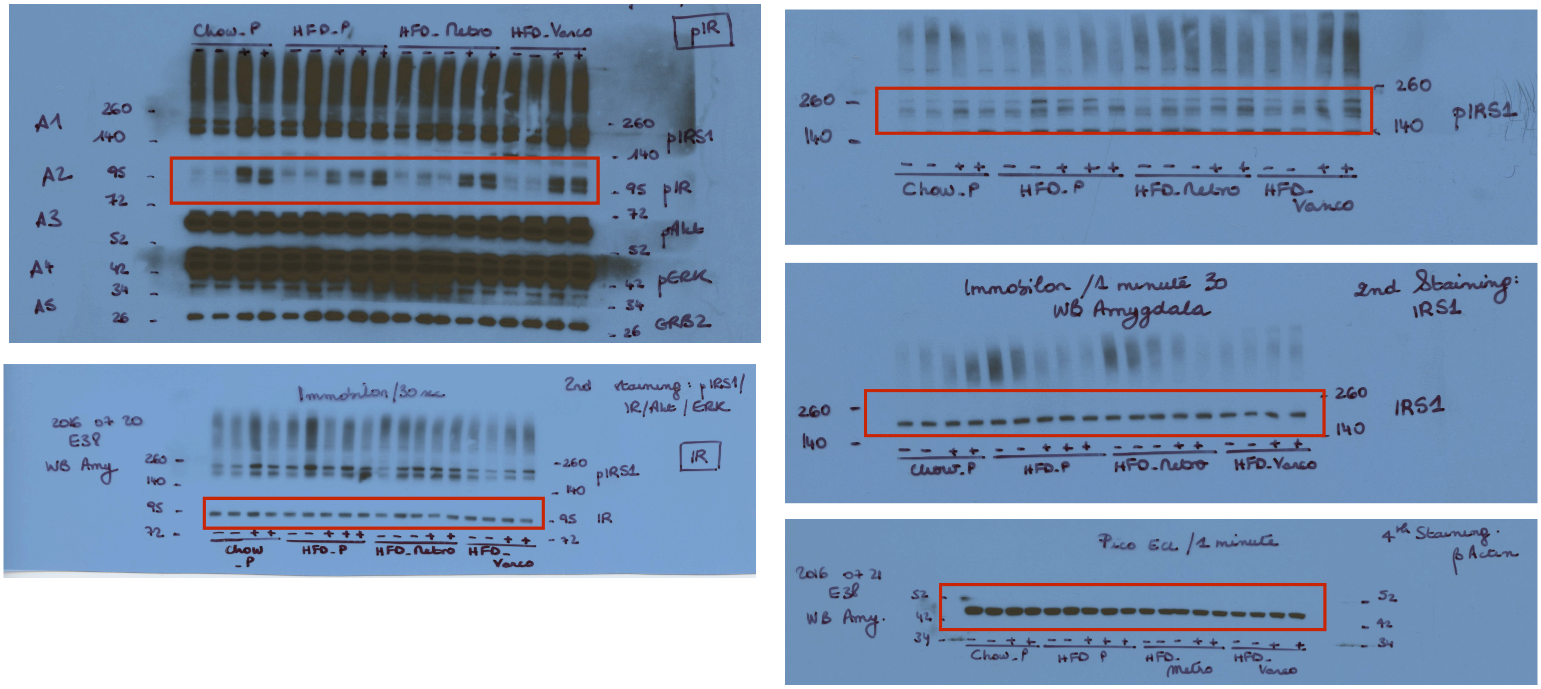

Figure S5a

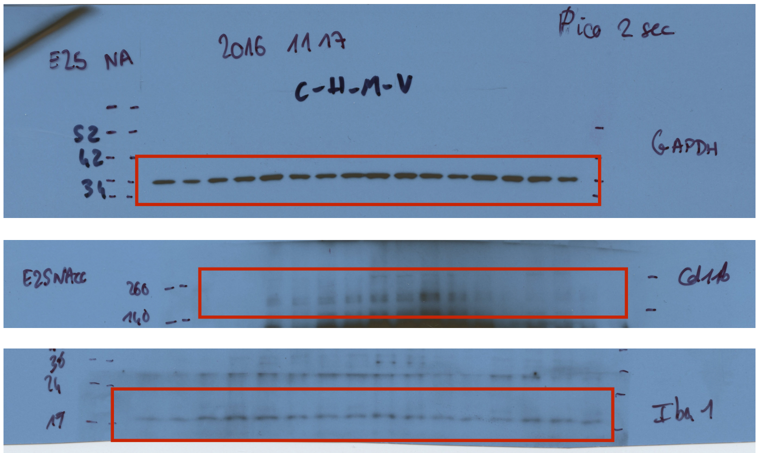

Supplementary Figure 10. Uncut blots. The red sections indicate blot results shown in the indicated figures.

Supplementary Figure 11

Figure S7b

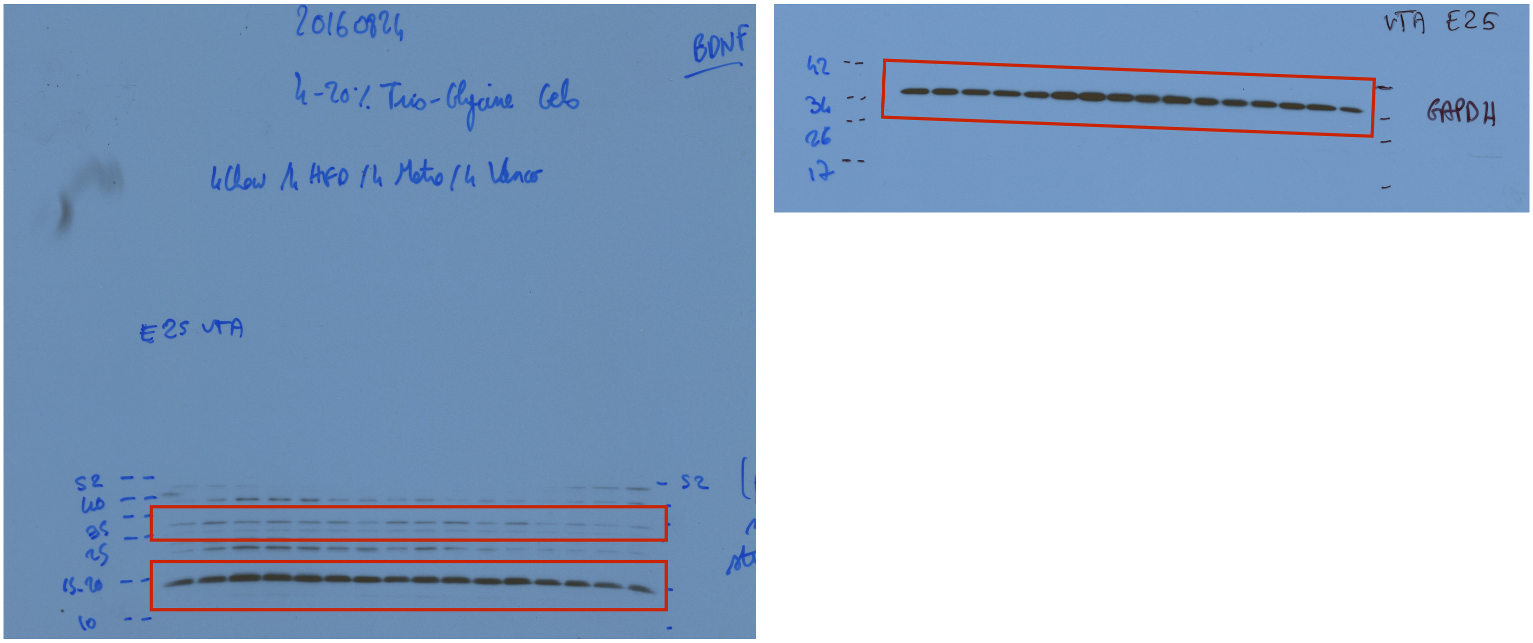

Supplementary Figure 11. Uncut blots. The red sections indicate blot results shown in the indicated figures.
